# Essential Oils as Alternative Biocides for the Preservation of Waterlogged Archaeological Wood

**DOI:** 10.3390/microorganisms8122015

**Published:** 2020-12-16

**Authors:** Federica Antonelli, Marco Bartolini, Marie-Laure Plissonnier, Alfonso Esposito, Giulia Galotta, Sandra Ricci, Barbara Davidde Petriaggi, Cristian Pedone, Antonella Di Giovanni, Silvano Piazza, Francesca Guerrieri, Manuela Romagnoli

**Affiliations:** 1 Department for Innovation in Biological, Agro-Food and Forestry Systems (DIBAF), Tuscia University, 01100 Viterbo, Italy; mroma@unitus.it; 2 Biology Laboratory, Istituto Centrale per il Restauro (ICR), Ministry of Cultural Heritage and Activities and Tourism (MIBACT), 00153 Rome, Italy; marco.bartolini@beniculturali.it (M.B.); giulia.galotta@beniculturali.it (G.G.); sandra.ricci@beniculturali.it (S.R.); 3 Epigenetics and Epigenomic of Hepatocellular Carcinoma, U1052, Cancer Research Center of Lyon (CRCL), 69424 Lyon CEDEX 03, France; marie-laure.plissonnier@inserm.fr; 4 Department of Cellular, Computational and Integrative Biology–CIBIO, University of Trento, 38123 Trento, Italy; alfonso.esposito@unitn.it (A.E.); silvano.piazza@icgeb.org (S.P.); 5 Underwater Archaeological Operations Unit, Istituto Centrale per il Restauro (ICR), Ministry of Cultural Heritage and Activities and Tourism (MIBACT), 00153 Rome, Italy; barbara.davidde@beniculturali.it; 6 Restoration Laboratory of Organic Excavation Materials, Istituto Centrale per il Restauro (ICR), Ministry of Cultural Heritage and Activities and Tourism (MIBACT), 00153 Rome, Italy; cristianpedone95@hotmail.com (C.P.); antonella.digiovanni@beniculturali.it (A.D.G.); 7 Computational Biology, International Centre for Genetic Engineering and Biotechnology, 34149 Trieste, Italy

**Keywords:** cinnamon, wild thyme, common thyme, cultural analyses, ATP bioluminescence, NGS, minimum inhibitory concentration (MIC), WAW, archaeological wood biodegradation, archaeological wood restoration

## Abstract

Waterlogged archaeological wood is exposed to a high risk of biological degradation during the post-excavation phases of storage and restoration. For this reason, often biocides must be used to preserve wooden remains. In the present work three essential oils (cinnamon, wild thyme, and common thyme) were tested as possible alternative biocides to use in the preservation of waterlogged archaeological wood. The oils were first tested in vitro to establish the minimum inhibitory concentration (MIC) and to evaluate the biocidal activity on selected fungal strains. Then, the established MIC was applied on waterlogged archaeological wood samples and during an actual restoration treatment. The effectiveness of the oils was evaluated through cultural analyses, ATP quantification, and next-generation sequencing. The results showed that the oils caused a significant decrease in the vitality of fungal mycelia grown in vitro and of the microbiota present in treated wood and storage water. Furthermore, an influence on the composition of the bacterial communities of treated wood samples was observed. Although further tests are needed to evaluate interferences with the materials used during restoration procedures, essential oils could be considered as a possible alternative to the currently used biocide.

## 1. Introduction

Aquatic and land waterlogged sites represent unique environments in which archaeological wood is protected from the fast biological decay occurring in terrestrial contexts thanks to the low oxygen level. In anaerobic or almost anaerobic conditions only slow degrading bacteria can attack wood. However, during the excavation, storage, and restoration operations, waterlogged archaeological wood (WAW) is exposed to higher oxygen levels and therefore it can undergo faster biological decay due to the action of fungi [1,2,3,4,5,6,7,8]. Fungal species able to degrade WAW are taxonomically classified in the phyla Ascomycota and Basidiomycota or are known as Deuteromycetes [1]. Through the action of extracellular enzymes that depolymerase the wood cell walls’ polymers (lignin, cellulose, and hemicelluloses), these microorganisms produce different decay forms that can be grouped in three main categories soft, brown, and white rot [1,2,3,4,9,10]. Microbial degradation patterns and cell wall polymers degradation have been widely studied and characterized through microscopic, chemical, and physical analyses [11,12,13,14,15,16,17].

Considering the biological threat WAW is exposed to after recovery, precautions must be taken to limit wood degradation. The supplement to the Official Gazette of the Italian Republic n. 244 (2001), also known as “Museum Standards”, and the EN16873:2016 [18] suggest the thermohygrometric values recommended for the prevention of microbiological attacks on organic artifacts. According to these laws, during the storage phases preceding the restoration WAW should be preserved completely submerged in water at a temperature lower than 4 °C. When it is not possible to respect these guidelines, a biocide should be used. Biocidal products are “active substances and preparations containing one or more active substance, […] intended to destroy, deter, render harmless, prevent the action of, or otherwise exert a controlling effect on any harmful organism by chemical or biological means” [19,20]. Nowadays the biocides most frequently used in the preservation of archaeological wood are chemical preservatives (e.g., quaternary ammonium salts, derivatives of the isothiazolone class, etc.) [21].

In recent years, more and more attention has been paid to the identification of biocides of natural origin. Several studies showed the effectiveness of substances extracted from plants and animals in limiting and/or contrasting the growth of organisms and microorganisms and their application has been proposed in different fields (e.g., food preservation, pharmaceutical). Even in the conservation of cultural heritage, several attempts have been made in exploring the biocidal potential of natural products for the control of biodegradation of textiles, stone, and wooden artifacts [22,23,24,25,26,27,28,29,30,31]. Anyway, these substances have never been used to protect WAW.

The International Organization for Standardization (ISO) defined essential oil (EO) as “product obtained from a natural raw material of plant origin, by steam distillation, by mechanical processes from the epicarp of citrus fruits, or by dry distillation, after separation of the aqueous phase—if any—by physical processes” [32]. EOs are volatile, complex compounds produced by plants as secondary metabolites. At room temperature, they are liquid, limpid, rarely colored, and soluble in lipid and organic solvents, generally with lower density than water. In nature, they play an essential role in protecting plants from bacterial, viral, fungal, and insect attacks as well as against herbivory. They can be present in all plant organs (e.g., buds, flowers, leaves, seeds, twigs, stems, fruits, bark) and are generally stored in secretory cells, cavities, canals, or glandular trichomes. The EOs producing plants are usually known for their numerous useful effects (e.g., antioxidant, analgesic, sedative, spasmolytic, local anesthetic) and are used for food preservation or as part of the traditional pharmacopoeia. The antimicrobial activity of these natural products has been widely studied and several of their biological effects (e.g., cytotoxicity, phototoxicity, mitochondrial damaging) are well known [33]. Due to the great number of constituents, EOs seem to have no specific cellular targets so they could be considered as biocides with broad-spectrum activities [34]. As lipophiles, they can pass through the cell wall and cytoplasmic membrane altering the polysaccharide layers structure and permeabilizing the membrane, but they can also target proteins, enzymes, and DNA [33,35,36].

Several works tested EOs and/or their components against wood degrading microorganisms [36,37,38,39,40,41,42,43]. Among the analyzed compounds, cinnamon and thyme EOs and their main components (i.e., cinnamaldehyde, eugenol, thymol, and carvacrol) proved to have a biocidal activity against several wood degraders. Cinnamon oil was tested against mold and sapstain fungi, proving to be highly effective when used in ethanol against brown and white rot fungi [44,45]. The essential oil of *Thymus vulgaris* has biocidal activity against bacteria isolated from deteriorated wood (*Bacillus subtilis* and *Bacillus safensis*). Singh and Chittenden [46] by screening 12 essential oils to evaluate their antifungal activity against common mold, stain, and wood decay fungi showed that eugenol and cinnamaldehyde were the most effective in inhibiting the growth of test fungi on treated wood blocks. Furthermore, isolated cinnamaldehyde, eugenol, thymol, and carvacrol proved to be highly effective against brown and white fungi [22,37,43,44,45,47,48].

Several techniques can be used to evaluate the biocidal activity of tested products, mainly depending on the selected product and the microorganisms to be devitalized (e.g., microbiological analyses, ATP quantification, fluorescence evaluation, and molecular techniques). Cultural tests are routinely used to evaluate the presence of waterlogged wood biodeteriogens while other techniques, like ATP quantification and next-generation sequencing (NGS) have seldom been applied to this field.

The quantification of ATP through bioluminescence is a valuable way to evaluate cell vitality. Here the luciferase enzyme, from the firefly of the genus *Photinus*, reacts with ATP in the presence of its substrate luciferin, oxygen, and magnesium ions to produce oxyluciferin, AMP (adenosine monophosphate), pyrophosphate, and light. The amount of light generated by this enzymatic reaction is proportional to the ATP in the reaction. The ATP quantification methods are rapid, robust, easy to perform, affordable, and can detect both cultivable and uncultivable organisms, with high sensitivity from samples with extremely low microbial burden [49,50,51]. ATP biomonitoring is widely used in the food processing industry for the detection of microbial contamination [52,53,54], in the healthcare sector to monitor the cleaning practices [55,56] and the microbiological quality of the water [57,58]. The quantification of ATP has also been applied in the field of the conservation of cultural heritage (stone monuments, paper, and textile) to survey the biological contamination and to evaluate the efficacy of biocidal treatments [59,60,61,62,63,64,65].

NGS refers to the newest molecular technologies relying on a combination of template preparation, sequencing and imaging, and data analysis [66]. Even if the application of NGS to the study of WAW is recent [67,68,69,70], the high-throughput sequencing of microbial genomic DNA by MiSeq gave interesting results highlighting the presence of potential biodeteriogen taxa [71].

The present work was aimed to find bioinspired preservatives that could substitute the biocides currently used during the storage and restoration of WAW. In particular, the study tested three essential oils from organic farming *Cinnamomum zeylanicum* Blume (cinnamon bark), *Thymus serpyllum* L. (wild thyme), and *Thymus vulgaris* L. (common thyme) and evaluated their biocidal activity comparing the results obtained by cultural analyses, ATP bioluminescence assays, and NGS.

The results showed that the hydroalcoholic solution containing the three tested oils, 1% in concentration, inhibit the spore germination and have a fungicidal effect on selected fungal strains in vitro. The use of this same concentration to preserve WAW submerged in water leads to a significant decrease in the vitality of the microorganisms present in the wood and the storage water and influences the composition of the bacterial communities.

## 2. Materials and Methods

### 2.1. Essential Oils

The three essential oils (EOs) from organic farming tested during the experimentation (cinnamon bark, wild thyme, and common thyme) were provided by Flora srl (Pisa, Italy).

Flora srl characterized the EOs composition through gas chromatography–mass spectrometry (GC–MS): instrument Clarus 500GC/FID/MS (Perkin Elmer, Waltham, MA, USA); column, 5% diphenyl + 95% dimethylpolysiloxano. The results are reported in Table 1.

### 2.2. Biocidal Activity Assays

The biocidal activity of the selected EOs was evaluated both in vitro and on the microbiota present in WAW samples.

Since the work aimed to find natural preservatives that could substitute the biocides currently used during the storage and restoration of WAW, the tests were performed using procedures as similar as possible to the application method to be used during these phases. For this reason, the oils were not tested as pure compounds but dissolved in ethanol.

For in vitro tests, EOs were diluted in a solution 50/50 *v*/*v* of deionized water and ethanol. While for the experiments carried out on WAW the oils were dissolved in an equal volume of ethanol and then added to the storage water or consolidation bath (for further details see next paragraphs).

From now on, C, WT, and CT refer to hydroalcoholic solution, respectively, of cinnamon, wild thyme, and common thyme oils or to these compounds dissolved in ethanol.

Biocidal activity was evaluated through cultural analyses, quantification of ATP bioluminescence, and/or by characterizing the microbiota present inside WAW through next-generation sequencing (NGS).

### 2.3. Fungal Strains

For in vitro tests, four cellulolytic fungal strains were selected: *Chaetomium* sp., *Fusarium* sp., *Aspergillus japonicus*, and *Stachybotrys chartarum*. Fungi were isolated from paper artifacts and identified to the genus or species level through appropriate identification keys (among the others [72,73]). The isolates were maintained on Malt Extract Agar (MEA) (Sigma-Aldrich, St. Louis, MO, USA), stored at 4 °C. Spore suspensions adjusted to the concentration 44–56 × 10^4^ spores/mL were obtained for each strain by mixing the spores with sterile saline solution containing 0.01% (*v*/*v*) Tween 20 to prevent spore aggregation. Spores concentration in the suspensions was quantified in a Thoma counting chamber.

### 2.4. In Vitro Tests

The effectiveness values of four different concentrations (3%, 1%, 0.7%, and 0.5%) of the selected EOs was estimated by minimum inhibitory concentration (MIC).

For each oil, the four fungal strains spore suspensions (1 mL) were uniformly distributed on a set of three Petri dishes (9 cm Ø) containing MEA. After the inoculum, 500 µL of biocidal solution was sprayed on the medium surface and the plates were incubated at 27 ± 2 °C. Control tests were carried out under the same condition by substituting the biocide with a solution 50/50 *v*/*v* of deionized water and ethanol (WE). To evaluate the spore vitality one Petri dish was inoculated with the spore suspension and incubated at the same temperature. Fungal growth was visually evaluated after 3, 6, and 10 days of incubation; the antifungal activity was assessed by the presence/absence of fungal growth.

The biocidal activity of the established MIC was evaluated on the mycelium of the same selected strains. For each strain, a Petri dish containing MEA was inoculated with fungal spores and incubated at 27 ± 2 °C for 3 days. The grown, not-sporigenous mycelia were sprayed with 1 mL of the solutions containing the MIC of the three oils. Control plates were treated with a solution of deionized water and ethanol (WE). The biocide efficacy was evaluated through cultural and biochemical tests. Seven days after the treatment, three small pieces of mycelium (about 10 mg each) were taken from each of the treated plates and transferred on a new culture medium. The plates were then incubated at 27 ± 2 °C. The biocidal activity was assessed basing on the presence/absence of fungal growth.

Biochemical tests involved the quantification of the ATP bioluminescence before the oil application and after 7 days from the treatment. To extract the ATP from fungal cells, 10 mg of mycelium were dispersed in 1 mL of deionized water containing sterile glass beads and vortexed for about 3 min. The fungal cells were then lysed with dimethyl sulphoxide (DMSO) at a concentration of 90% in TAE buffer (a buffer solution containing a mixture of trishydroxymethyl-aminomethane base, acetic acid, and ethylene diamine tetra-acetic acid), at pH 7.75. DMSO was preheated in a sterile 1.5 mL Eppendorf tube at 100 °C for 2 min, after adding the fungal suspension it was incubated for 1 min at 100 °C and then rapidly cooled in an ice-bath, as described by Rakotonirainy et al. [62]. The ATP was extracted in a reaction volume of 100 µL containing 20 µL of mycelium suspension and 80 µL of DMSO. The cellular ATP bioluminescence was evaluated by adding to the lysate 100 µL of detection reagent obtained by mixing the ATP Water-Glo^®^ Substrate CS193119 (Promega, Madison, WI, USA), provided as a powder, and ATP Water-Glo^®^ Reconstitution Buffer CS 193109 (Promega).

Free ATP was quantified replacing DMSO with the same volume of sterile saline solution. Blank tests were performed by substituting the sample with the same volume of extraction reagent (for cellular ATP tests) or of saline sterile solution (in the case of free ATP). The cellular ATP values reported in the results were obtained subtracting the free ATP and the blank values to the registered cellular ATP. All the tests were performed on three aliquots of the sample, and the results are reported as the average value of the three readings. The bioluminescence was registered as relative light unit (RLU) through a luminometer GloMax^®^ 20/20 (Promega) combined with GloMax^®^ Spreadsheet Interface Software (GLOMAX SIS v1.10.0) using the default setting.

The possible influence of the EOs on the ATP quantification was evaluated by adding 1 µL of the three EOs to 99 µL of standard ATP (REF: F203A, Promega) at concentration 10,000 pg/mL. The registered RLUs were compared to those obtained by substituting the oils with 1 µL of sterile deionized water.

### 2.5. Tests on the Waterlogged Archaeological Wood Microbiota

To evaluate the biocidal activity of the oils against the microbiota present inside WAW, the EOs MIC dissolved in ethanol was first tested on small samples of WAW and then the best performing EO was used as preservative during the restoration of archaeological wooden poles.

Five WAW samples (3.0 × 1.5 × 1.5 cm) were obtained from the remains pertaining to a Roman shipwreck dated back to the end of the 2nd century AD, recovered in 2015 from the site of the ancient port of Neapolis (Naples, Campanian region, Italy) [74], and stored in water at 4 °C, in the dark until the moment of the test. Wood was cut with a sterile blade and the samples were placed in sterile containers with 20 mL of the water in which the remains were stored until the test began. Three of the five subsamples were treated with C, WT, and CT. A total of 200 µL of the EOs dissolved in an equal amount of ethanol was added to the storage water. The fourth fragment was treated with the same amount of ethanol used to dissolve the oils (E). The last subsample was kept as untreated control (NT). The biocidal activity was evaluated through the quantification of ATP bioluminescence and by examining the total genomic DNA extracted from the wood. All tests were carried out after 1 month from the beginning of the treatment.

The biochemical tests were carried out both on the storage water and the wood. For the tests on water, the lysis and detection procedures described in Section 2.4 were followed. The20 µL of fungal suspension was substituted with the same amount of storage water. For the tests carried out on wood, 100 mg of wood was added to 1 mL of sterile water containing sterile glass beads and vortexed for about 3 min. The microbial cells extracted from wood were lysate and the ATP was quantified as described in Section 2.4. To evaluate the possible influence of wood extractives on the ATP quantification, 20 µL of the suspension obtained by vortexing the wood were added to 80 µL of standard ATP at concentration 10,000 pg/mL. The registered RLUs were compared to those obtained by substituting the suspension with 20 µL of sterile deionized water. Considering a possible variability in the amount of extractives present in the suspension after wood vortexing, the tests were carried out on all the lysate suspensions.

To evaluate the effect of the essential oils on the microbiota composition, NGS was performed on the treated and untreated wood (three replicates for each sample) and on the storage water (one replicate). Microbial DNA was extracted from 100 mg of wood frozen in liquid nitrogen and homogenized using mortar and pestle, and from 100 µL of storage water. Samples were incubated for 5 min at 95 °C in a Lysis buffer (A509C, Promega) modified with 3% of polyvinylpyrrolidone (PVP). Proteinase K (MC5005, Promega) was added to the buffer and the samples were incubated for 25 min at 65 °C and centrifuged at 13,000 rpm. The samples were then processed by the kit Maxwell^®^ RSC Plant DNA Kit (AS1490, Promega) and total genomic microbial DNA was extracted with Maxwell^®^ RSC Instrument (Promega). V3-V4 region of 16S rRNA gene (amplified using the primers described in Illumina 16S protocol: # 15044223 Rev. B) and ITS2-rDNA fungal subregion (amplified using the following primers: ITS3 PCR Forward Primer 5′ TCGTCGGCAGCGTCAGATGTGTATAAGAGACAG-GCATCGATGAAGAACGCAGC-3′ and ITS4 Reverse Primer 5′ GTCTCGTGGGCTCGGAG-ATGTGTATAAGAGACAGTCCTCCGCTTATTGATATGC-3′) were subject to amplicon library preparation (according to Illumina’s instructions, 16S Metagenomic Sequencing Library Preparation, Part # 15044223 Rev. B). Sequencing was performed by Miseq (2 × 300 paired-end, 600-cycle) Illumina platform (Illumina, San Diego, CA, USA).

Raw fastq files were imported for preprocessing and OTU picking into qiime2 [75], the quality-check and clustering were done using DADA2 [76], representative sequences were aligned using mafft [77], uninformative positions were masked, and a phylogenetic tree was built with fasttree [78]. Taxonomic assignment for 16S rRNA and ITS data were performed using the SILVA and UNITE database [79,80]. Files containing the OTU tables, the phylogenetic trees, and the metadata, along with the beta-diversity distance matrices, were exported from qiime2 and imported into calypso for downstream analyses [81]. To determine the taxa which had differential abundance among the treatment we used the linear discriminant analysis (LDA) effect size (LEfSe) [82]. Alpha and beta diversity values were calculated in qiime and plotted using the calypso interface.

Data are available in SRA database (project PRJNA679775, in particular 16S data are available in SUB8581970 and ITS2 data are available in SUB8582450).

Cinnamon essential oil, selected based on the results obtained in the previous experimentations, was tested as preservative during one of the phases of the restoration project entitled “Restauro del relitto E dagli scavi del porto antico di Napoli. Due metodi di consolidamento a confronto” [83]. The tests were performed on wooden poles recovered from the excavation area of Piazza Municipio in Naples. The poles were treated into four tanks containing a lactitol-trehalose consolidation bath. Two of the tanks were transparent, the others opaque. After 1 month from the beginning of the consolidation treatment a conspicuous microbial growth was observed in all the containers, so it was decided to treat the baths with a biocide. Two of the tanks were treated with cinnamon. The amount of oil added to the baths was that necessary to obtain a final oil concentration of 1%. The oil was previously dissolved in an equal amount of ethanol.

The effect of the EO was compared to that of a usually used biocide, Preventol^®^ RI80, added to the two remaining tanks, 1% in concentration. The experimentation details are presented in Table 2.

The biocidal effect of C and Preventol^®^ RI80 was evaluated through biochemical tests and cultural analyses performed before the application of the biocides and after 1 week.

ATP bioluminescence was quantified on an aliquot of the impregnation baths of the four tanks. Lysis and detection procedures described in Section 2.4. were followed, the 20 µL of fungal suspension were substituted with the same amount of the impregnation baths.

Two tests were carried out to evaluate the possible influence of lactitol-trehalose and of Preventol^®^ RI80 on the ATP quantification. First, 20 µL of lactitol-trehalose solution (concentration 25%, as for the impregnation bath) were added to 80 µL of standard ATP at concentration 10,000 pg/mL. Secondly, 20 µL of the impregnation bath treated with 1% of Preventol^®^ RI80 were added to 80 µL of standard ATP at the same concentration. The registered RLUs were compared to those obtained by substituting the solution and bath aliquots with 20 µL of sterile deionized water.

Cultural analyses were carried out on the same days of the biochemical tests. A stock solution was prepared by adding 1 mL of impregnation bath to 9 mL of sterile saline solution. For the prebiocide test, 1 mL of the dilutions 10^−4^–10^−6^ were inoculated on MEA in duplicate. For the tests performed after the application of the biocides, based on the results of ATP quantification it was decided to plate the stock solution and the 10^−1^ dilution for the Preventol^®^ treated tanks and the 10^−2^ and 10^−3^ dilutions for C treated baths. The plates were incubated at 27 ± 2 °C and the colonies were counted after 3, 7, and 14 days. The results are reported as CFU/mL.

## 3. Results

### 3.1. In Vitro Tests

Tests carried out in vitro allowed evaluating the effect of the three tested essential oils (EOs) on the fungal spore germination and on the mycelium vitality.

Results (Table 3) showed that the hydroalcoholic solution containing cinnamon oil 0.5% in concentration was effective against all the fungal strains except for *Aspergillus japonicus*, while the same concentration of common thyme and wild thyme was effective only against *Stachybotrys chartarum*. The concentration 0.7% gave good results against *Chaetomium* sp. and *S. chartarum* for the two thymes but only against *A. japonicus* for cinnamon. The 1% and 3% concentrations of the three EOs were efficient against all the strains so the MIC for the three EOs was set at 1%.

**Figure 1:**
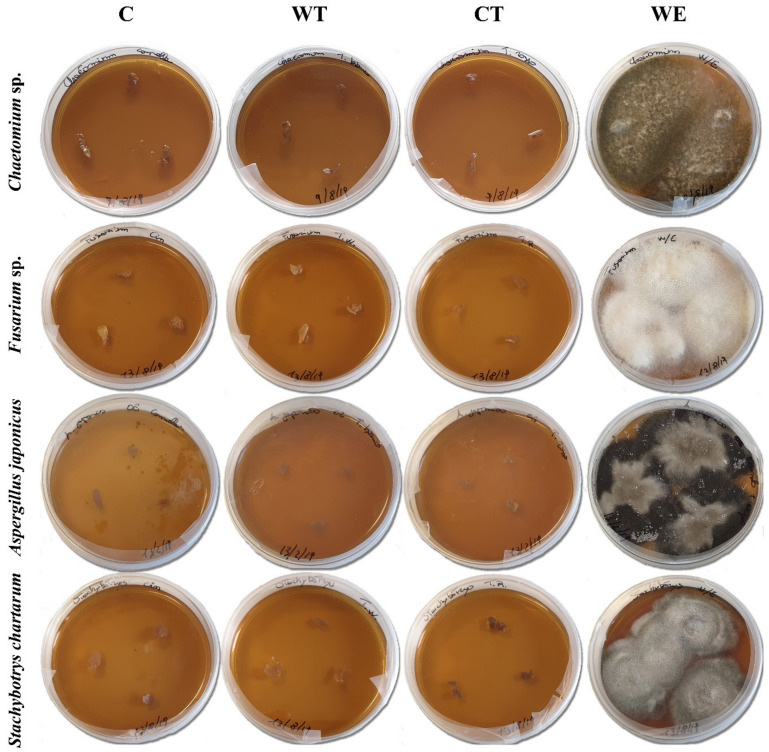
Results of cultural tests carried out on the fungal mycelia treated with the EOs MIC. (C: cinnamon; WT: wild thyme; CT: common thyme; WE: water-ethanol).

ATP bioluminescence assays allowed to evaluate the biocidal activity of the established MIC on the mycelium of the selected fungal strains. As the results clearly show (Table 4) the hydroalcoholic solutions containing the three oils allowed obtaining a significant decrease of the fungal vitality of all the strains. C and WT caused an ATP decrease equal to 99.9% or 100% in all the tests. ATP decrease registered for CT was 100% for *Chaetomium* and *Fusarium*, 99.9% for *A. japonicus*, and 98% for *Stachybotrys*. The results of cultural analyses showed that all the pieces of mycelium transferred on the new culture medium were not able to grow (Figure 1).

The results of biochemical analyses obtained for the control tests carried out treating the mycelium with a solution of water and ethanol (WE) varied greatly depending on the fungal strain. For *Chaetomium* and *S. chartarum* 99.9% and 93% decreases were registered, respectively, while the vitality of *A. japonicus* was reduced by 37%. Instead, for *Fusarium* the amount of ATP registered after the treatment was higher respect to the pretreatment value. Despite the contrasting biochemical results, the outcomes of cultural analyses were more homogeneous and showed that all the WE treated mycelia when transferred on a new culture medium were still able to grow (Figure 1).

The comparison of biochemical and cultural results obtained for C, WT, CT, and controls clearly demonstrates that while ethanol had a fungistatic effect, the solutions containing EOs can be considered as fungicides.

The tests carried out to evaluate the possible influence of the EOs on the ATP quantification showed that none of the three oils had a registrable effect. The standard ATP values registered with and without the oils’ addition were comparable, a variation of ±1% respect to the pure standard was observed. This range of variation is the one usually observed in a set of repeated measures on the standard and so it can be considered as irrelevant.

### 3.2. Waterlogged Archaeological Wood Samples

The study carried out on WAW samples allowed to quantify the biocidal effect of the EOs MIC on the microbial communities present in both the storage water and the wood and to evaluate the influence of tested biocides on the bacterial and fungal communities.

Biochemical tests showed that the addition of the three ethanol dissolved oils to the storage water caused a decrease in the microbial vitality compared to the untreated control sample (Table 5). Tests carried out to evaluate the possible influence of wood extractives on ATP quantification showed that the presence of these compounds in the lysate suspension caused a decrease ranging between 15% and 20% of the standard ATP values. Therefore, correction factors were calculated and applied to the data. Only the corrected results are shown in the text.

The best results were obtained for C. A percent decrease of 100% on water and 99.9% on wood was registered from the comparison of ATP values of this oil’s solution with untreated control. WT produced a vitality decrease by 89% on water and 96.7% on wood. The worst results were obtained with CT, for this oil’s solution the residual microbial vitality was 1.5% for wood and almost 27% for water.

Control tests carried out on the ethanol treated sample showed that the ATP values registered for both water and wood were higher with respect to the untreated control. These data confirm what was observed in in vitro tests, ethanol has fungistatic but not fungicidal effect.

NGS analyses performed on treated and untreated wood and storage water allowed to evaluate the effect of the tested oils on the microbiota composition. The number of reads ranged from 28,233 to 180,107 (with an average of 92.685.6) for 16S rRNA, and 2.089–199.034 (with an average of 44,635.9) for ITS data (Table S1). The number of resulting OTUs ranged from 292 to 1106 (with an average of 594.8) for 16rRNA, and 24–130 (with an average of 65.6) for ITS (Table S1).

**Figure 2:**
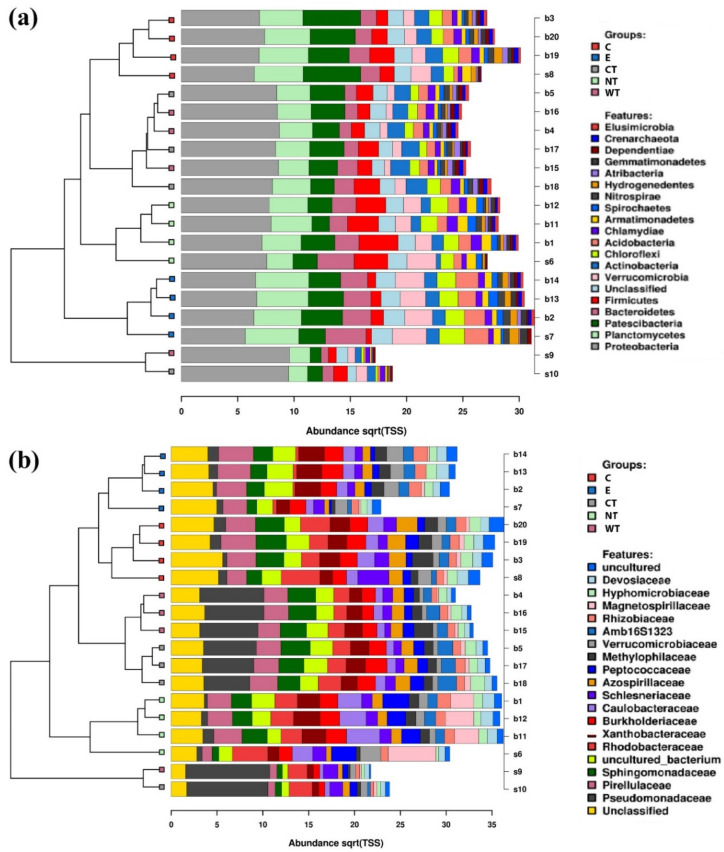
Stacked barcharts showing the relative abundance of bacterial phyla (**a**) and families (**b**) in each sample. Tips of the clustering tree are colored according to the treatments. (C: cinnamon; E: ethanol; CT: common thyme; NT: not-treated control; WT: wild thyme).

**Figure 3:**
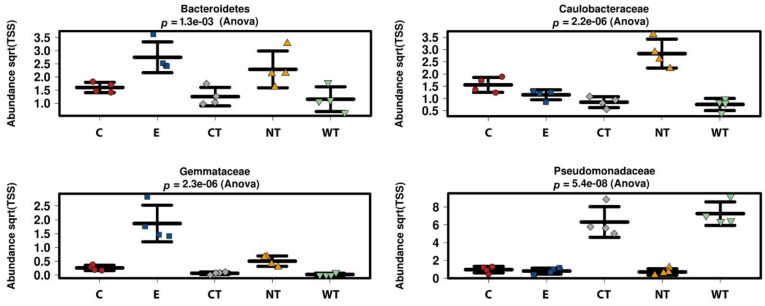
Boxplots showing the relative abundance of selected bacterial taxa identified as biomarkers in the LEfSe analysis. (C: cinnamon; E: ethanol; CT: common thyme; NT: not-treated control; WT: wild thyme).

At phylum level, it can be observed that Bacteroidetes bacteria are scarcely represented in all the oil treated samples (Figure 2a). In fact, while in controls (both E and NT) the mean relative abundance of this phylum is higher than 2 and exceeds 3.5 in two replicas, in treated samples it never exceeds 2, with a mean value equal to or lower than 1.5. A similar trend was observed for the phylum Verrucomicrobia, particularly underrepresented in the samples treated with the thyme oils (Figure S4). Observing the taxa abundances at family level (Figure 2b), similar trends were observed for Gemmataceae, Opitutaceae, Pedosphaeraceae, and Phaselicystidaceae (Figure S4). Instead, Caulobacteraceae (Figure 3), Magnetospirillaceae (Figure S4), and Rhodospirillaceae were enriched in untreated samples, while Gemmataceae (Figure 3) reported high relative abundances only in ethanol-treated wood and water. Finally, it is interesting to note that Pseudomonadaceae was enriched in T samples (mean relative abundances >6) while it presented abundances lower than 1 in all other analyzed samples (Figure 3).

**Figure 4:**
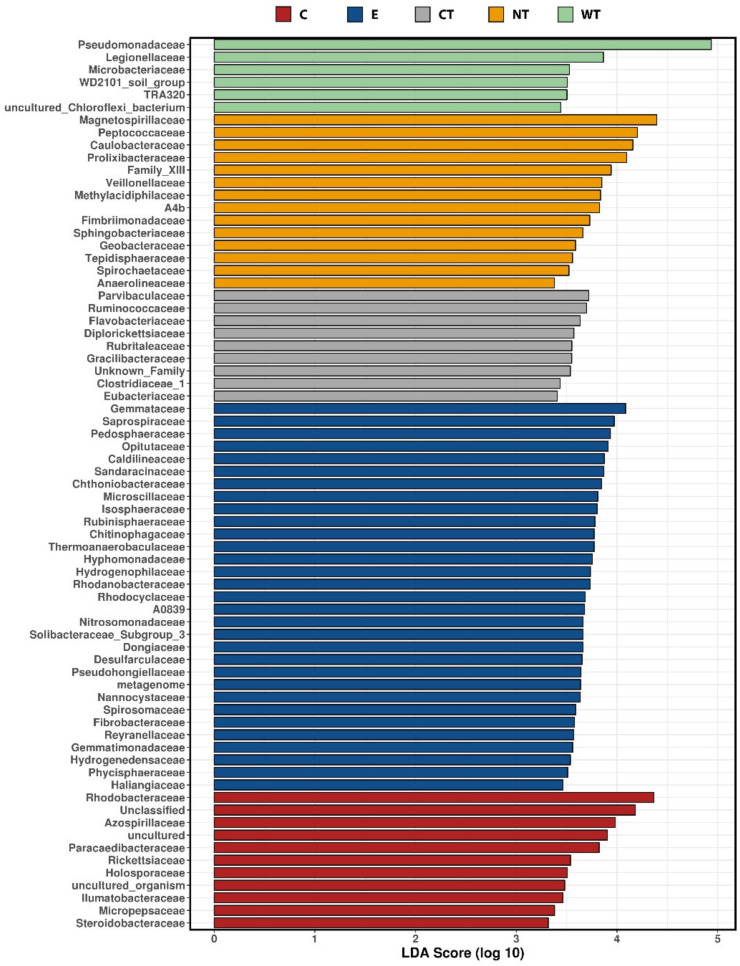
Taxa identified as biomarkers for each treatment by LEfSe analysis, LDA scores (log10) threshold was set to 3. (C: cinnamon; E: ethanol; CT: common thyme; NT: not-treated control; WT: wild thyme).

LEfSe analyses compared the relative abundances of the most represented bacterial families for each treatment. Taxa characterized by the highest LDA scores can be considered as the most resistant to the biocidal treatment. What is observed in Figure 4 confirms the data reported in Figure 2 and Figure 3, the most resistant taxa are the most represented in the bacterial communities of wood and water.

**Figure 5:**
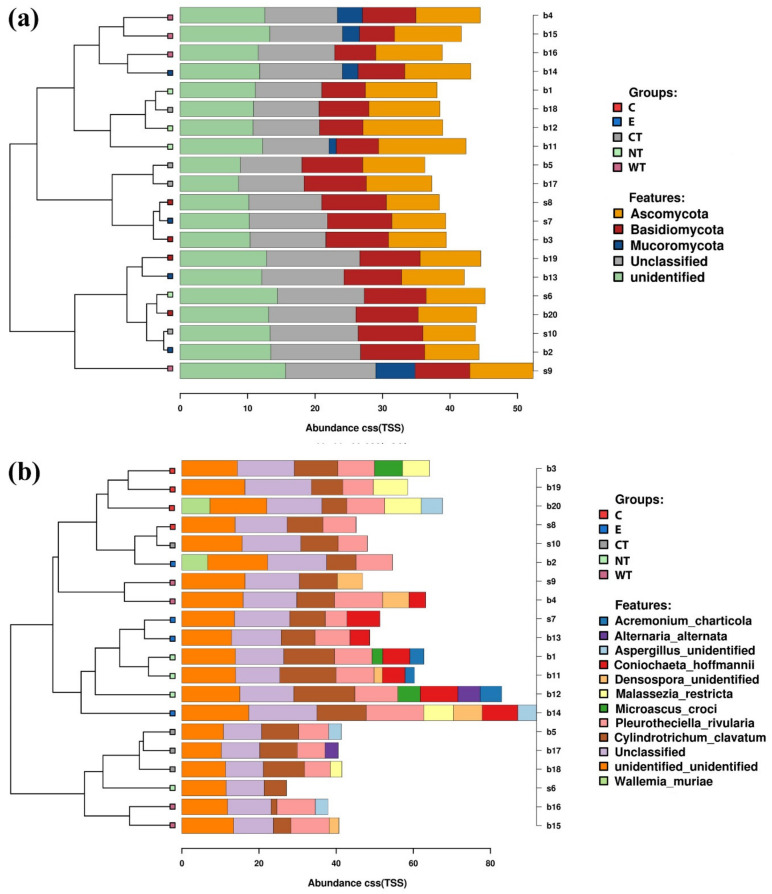
Stacked barcharts showing the relative abundance of fungal phyla (**a**) and species (**b**) in each sample. Tips of the clustering tree are colored according to the treatments. (C: cinnamon; E: ethanol; CT: common thyme; NT: not-treated control; WT: wild thyme).

**Figure 6:**
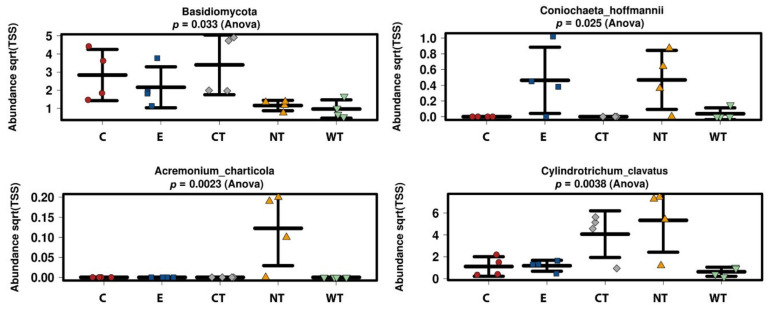
Boxplots showing the relative abundance of selected fungal taxa identified as biomarkers in the LEfSe analysis. (C: cinnamon; E: ethanol; CT: common thyme; NT: not-treated control; WT: wild thyme).

Differently from what was observed for the bacteria, fungal communities present in the treated and untreated samples are more similar (Figure 5 and Figure 6, and Figures S5–S8). Observing the relative abundances at phylum level (Figure 5a and Figure 6), it can be noticed that Basidiomycota was more represented in the C and CT treated samples and in E controls. This suggests that oils and ethanol were not efficient against the fungal taxa belonging to this phylum that were enriched after 1 month of treatment.

Regarding the identified fungal species (Figure 5b), no significant differences in terms of fungal community composition could be highlighted among the differently treated samples. The only species whose distribution could be attributed to the treatment are *Coniochaeta hoffmannii*, *Acremonium charticola*, and *Cylindrotrichum clavatus* (Figure 6)*. C. hoffmannii* was enriched in the control samples, both E and NT, and *A. charticola* was present only in untreated samples. *C. clavatus* was present with mean abundances higher than 4 in CT and NT while in the other samples its mean abundances were always lower than 1.

**Figure 7:**
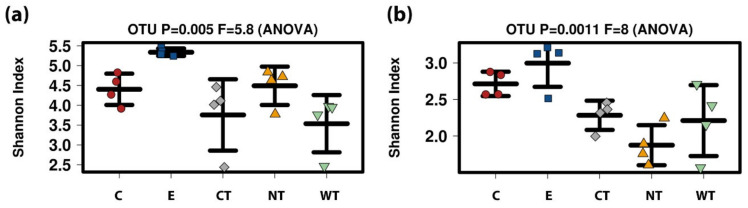
Boxplots of alpha-diversity index (calculated as Shannon index) (**a**) 16S. (**b**) ITS. (C: cinnamon; E: ethanol; CT: common thyme; NT: not-treated control; WT: wild thyme).

For both 16S and ITS, E samples were characterized by greater diversity (Figure 7). For 16S no significant differences were registered among the EOs treatments and the untreated control while for ITS C showed a slightly higher diversity with respect to CT and NT (*p*-values 0.016 and 0.49, respectively, after *t*-test) (Table S2).

**Figure 8:**
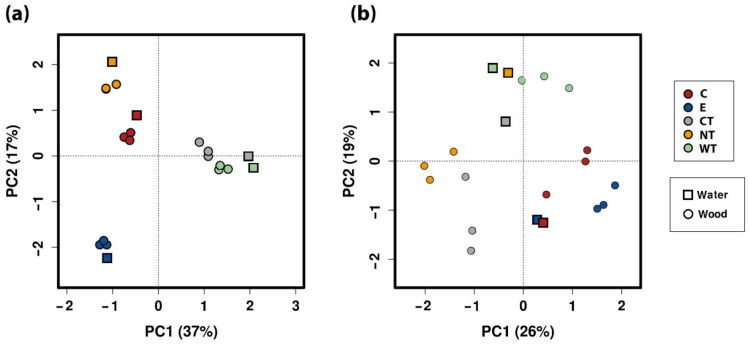
Principal component analysis calculated on the 16S (**a**) and ITS (**b**) ecological matrices at the level of OTU. (C: cinnamon; E: ethanol; CT: common thyme; NT: not-treated control; WT: wild thyme).

The principal component analysis (PCA) calculated on the ecological matrix for 16S at the level of OTU (Figure 8a) well separated the EOs treated samples and the controls while in the PCA calculated for ITS (Figure 8b) a less significant difference was observed. It is interesting to note that for ITS the water sample was always distant from the wood samples related to the same treatment.

### 3.3. Waterlogged Archaeological Wood Remains

The test performed during the consolidation treatment of the archaeological wooden poles allowed evaluating the biocidal effect of cinnamon essential oil during an actual restoration procedure and to compare it to a biocide currently used in the restoration laboratories.

The results of biochemical tests (Table 6) and cultural analyses (Table 7) showed that a marked decrease in the microbial vitality and in the contamination of the impregnation baths was obtained with both biocides. Of note, the tests carried out to evaluate the possible influence of lactitol-trehalose and of Preventol^®^ RI80 on the ATP quantification showed no remarkable effect. The standard ATP values registered in the presence of the mixtures lactitol-trehalose and lactitol-trehalose-Preventol^®^ RI80 and without them were comparable. The observed variation with respect to the pure standard measures is comparable to that usually observed in a set of repeated measures on the standard and so it can be considered as irrelevant.

The biochemical and microbiological results obtained with Preventol^®^ RI80 are slightly better with respect to cinnamon oil (Table 6, Table 7 and Table 8). For the commercial biocide, the ATP decrease was higher than 99.7% for both tanks and the CFU decreased by 99.9% in tank V2 and by 100% in tank V3. Instead, for C a residual ATP value corresponding to ca 1% of the pretreatment quantity was registered for both tanks and only in V4 a CFU decrease higher than 99% was observed.

## 4. Discussion

The results of the tests showed that the three EOs have biocidal activity both in vitro and on the microbiota present in WAW and in storage water.

In vitro tests proved that 1% of the oils dissolved in hydroalcoholic solution have a fungicidal effect on all the selected fungal strains. The experimentations carried out on WAW showed that the addition of the oils’ MIC dissolved in ethanol to the storage water or impregnation bath can lead to a decrease in the microorganisms’ vitality always higher than 73%, reaching 99–100% for cinnamon oil. The use of NGS on wood and water samples allowed evaluating the effect of the oils on the microbial communities. The differences observed in the composition of the bacterial communities present in the differently treated samples showed that the oils had a differential effect on the bacterial taxa present in wood and water. The three EOs had biocidal activity on Caulobacteraceae, Magnetospirillaceae, Rhodospirillaceae, and Gemmataceae, enriched only in the control samples, while the thyme oils were not effective against the taxa belonging to the family Pseudomonadaceae. The effect of the oils on the fungal communities was more homogeneous and only for few species (i.e., *Coniochaeta hoffmannii*, *Acremonium charticola*, and *Cylindrotrichum clavatus*) a differential distribution, linked to the treatment, was observed.

The higher diversity in the ethanol treated samples may be due to its toxic effect on some of the more abundant species that results in the emergence of more species belonging to the low-abundance tail. It has been shown elsewhere that intermediate disturbances (such as a 50% ethanol solution could be) increase microbial diversity [84].

Differences between the effect of the oils in vitro and on the wood were observed. In fact, while for in vitro tests the vitality decrease was close to 100% for all hydroalcoholic oils’ solutions on all fungal strains, with the only exception of CT on *S. chartarum*, in tests on storage water and wood only cinnamon oil allowed to obtain a difference in the ATP values reaching almost 100% with respect to untreated control.

A possible explanation is linked to the microbial communities present in the wood. The in vitro tests showed that the oils are effective against isolated strains, but their effectiveness could decrease in presence of a complex community. Molecular investigations carried out on the untreated WAW sample showed that the wood is characterized by the presence of several bacterial and fungal taxa. Furthermore, the fungal species present in wood and storage water were different from those used for in vitro experimentation and the effectiveness of the EOs tested against these strains may be different.

Surely, to explain the obtained results, the chemical composition of the oils must be taken into consideration. As shown in Table 1, the tested EOs are a complex mixture of molecules (mainly terpenes, terpenoids, and aromatic and/or aliphatic constituents characterized by low molecular weight). These molecules have a variety of targets in the microbial cells (mainly the membrane and the cytoplasm proteins) and can inhibit or slow the growth of bacteria and fungi, sometimes causing a complete alteration of the cell morphology [33,35,36]. Several studies, mainly conducted on human pathogens, analyzed the effect of the EOs and/or their constituents against microbial cells showing that usually the EOs biological effect is determined by the major components [85]. The cinnamon essential oil used in the experimentation is mainly composed by cinnamaldehyde, eugenol, caryophyllene, and linalool. The wild thyme oil is dominated by carvacrol, linalool, and y-terpinene while the common thyme is mainly composed of thymol, *p*-cymene, y-terpinene, and linalool.

Phenylpropenes (cinnamaldehyde and eugenol) are derivatives of cinnamic acid, characterized by an aromatic phenol group and a three-carbon propene tail. Eugenol interacts with bacterial cell membranes affecting the transport of ions and ATP and inhibits different bacterial enzymes like ATPase, amylase, and protease [86,87,88]. Furthermore, it is considered a growth inhibitor in fungi, it limits protein production and DNA replication and causes the lysis of spores [89,90]. The effect of cinnamaldehyde is linked to its concentration. A small amount of the molecules inhibits enzymes involved in important cell functions, at higher concentrations cinnamaldehyde is an ATPase inhibitor. Lethal concentrations of this molecule perturb cell membranes [35]. Tests carried out on wood decay fungi showed that cinnamaldehyde and eugenol have a synergistic effect being their combination effective at much lower concentrations than the molecules used alone [42,91].

The antimicrobial activity of terpenoids (*p*-cymene, y-terpinene, linalool, carvacrol, and thymol) is mainly linked to their functional groups, the hydroxyl group and the presence of delocalized electrons is particularly important for phenolic terpenoids. P-cymene, a monoterpene with a benzene ring without functional groups, can perturb microbial membranes, causing their expansion and a modification of membrane potential [92]. It does not directly affect the membrane permeability but can decrease its enthalpy and melting temperature [93]. Several tests showed that p-cymene is less effective than other terpenoids, but it can increase the antimicrobial activity of other compounds, like carvacrol [92,94,95,96]. Linalool is a terpene alcohol, able to cause a rise in intracellular cAMP [97]. Thymol is a phenolic monoterpenoid structurally similar to carvacrol but having a hydroxyl group at a distinct position on the phenolic ring. Thymol interacts with microbial outer and inner membranes causing structural and functional alterations (i.e., increase of permeability, release of K+ ions and ATP, release of lipopolysaccharides) and can interact with membrane proteins and intracellular targets [98,99,100,101]. The phenolic monoterpenoid carvacrol, like thymol, alters the structure and function of cell membranes, causing an increase in permeability [93,98,100,102]. Furthermore, carvacrol is one of the few components of EOs able to disintegrate the outer membrane of Gram-negative bacteria [103]. Both thymol and carvacrol have an antifungal activity linked to the formation of lesions in the cytoplasmic membranes and a reduction in ergosterol content [104]. Compared to others terpenoids present in EOs, carvacrol is a more effective antimicrobial agent [92,94,95].

As said, biophysical and biological features of an EO reflect those of its major components. Anyway, even if no data are available on the biological activity of other minor components, studies evidenced that possibly the activity of the most abundant molecules may be modulated by the minor ones [105,106,107].

Considering the results obtained in the present experiment, we can assess that the synergic action of cinnamaldehyde-eugenol present in cinnamon oil is more effective in devitalizing the microbiota present in wood and storage water with respect to the molecule combinations of thyme oils. Furthermore, the common thyme composition has greater antimicrobial activity than the wild thyme one despite the greater antimicrobial effect reported for carvacrol, of which this oil is enriched, compared to the other terpenoids.

Finally, it cannot be excluded a possible influence of the setting up of the experimentations on the results. During the tests performed on WAW samples, the same amount of ethanol was used for the three oils. Perhaps, this quantity was not sufficient to completely suspend wild and common thyme and this could have led to a lower effectiveness. For the tests carried out during the consolidation of the archaeological wooden poles an amount of ethanol equal to the oil volume was added to cinnamon EO before its addition to the consolidation bath. Possibly, this volume was not sufficient to correctly dissolve the EO in the tank’s water. Furthermore, it must be considered that a possible interaction with the consolidants present in the bath might have decreased the biocidal effect of the oil. More tests are needed to evaluate the actual influence of the quantity of ethanol used to dissolve the oil and the presence of consolidants.

Of note, in view of an application of cinnamon oil as a preservative in the field of restoration of WAW, it must be taken into account that the oil has a solvent effect on some plastic materials. In fact, during the experimentation the pumps used for the bath recirculation and the plastic accessories of the instruments used to evaluate the density of the suspensions suffered damage due to the presence of the oil (Figure S9).

## 5. Conclusions

The present work proved that the three tested essential oils, cinnamon, wild thyme, and common thyme, dissolved in ethanol have a biocidal effect both in vitro, on the selected fungal strains, and on the microbiota present in WAW. The results obtained for the three oils were slightly different, with cinnamon being the most effective. In fact, the addition of 1% of this EO dissolved in ethanol to the wood storage water allows obtaining a decrease by almost 100% of the vitality of the microbiota present inside the wood. Furthermore, the NGS analyses showed that the oils affect the composition of WAW bacterial communities having a differential effect on the bacterial taxa.

In conclusion, even if further experiments should be planned to set a protocol of application and to evaluate the interactions of the EOs with plastic materials, the good results obtained in testing the oils as alternative biocides for the preservation of WAW revealed the potential of these products during the delicate conservation and restoration phases of wooden artifacts.

## Figures and Tables

**Table 1:** Composition of the essential oils (EOs) selected for the experimentation.

Cinnamon		Wild Thyme		Common Thyme	
Component	%	Component	%	Component	%
α-pinene	0.67	α-thujene	1.02	Canfene	1.09
Canfene	0.25	α-pinene	0.76	α-pinene	0.66
β-pinene	0.26	Sabinene	0.28	α-thujene	1.21
α-phellandrene	1.09	β-myrcene	1.42	β-pinene	0.15
α-terpinene	0.06	α-terpinene	1.19	β-myrcene	1.76
p-cymene	1.54	p-cymene	5.24	a-phellandrene	0.16
Limonene	0.55	Limonene	0.25	α-terpinene	1.24
β-phellandrene	1.88	β-phellandrene	0.23	Limonene	0.49
Linalool	3.61	1.8 cineol	0.33	1.8 cineol + β-phellandrene	0.27
Hydrocinnamaldehyde	0.40	y-terpinene	4.02	y-terpinene	9.85
c-cinnamaldehyde	0.36	c-sabinene hydrate	0.46	p-cymene	17.69
o-anisaldehyde	0.07	Linalool	8.22	Terpinolene	0.11
t-cinnamaldheyde	69.81	Borneol	0.42	t-thujanol	0.25
Cinnamic alcohol	0.01	Terpinen-4-olo	0.62	Camphora	0.81
Eugenol	3.05	α-terpineol	0.61	Linalool	4.71
Hydrocinnamil acetate	0.07	Thymol	2.59	β-caryophyllene	2.29
α-copaene	0.63	Carvacrol	68.49	Neral	0.08
β-caryophyllene	5.58	β-caryophyllene	0.94	α-terpineol	0.12
Cinnamil acetate	2.04			Borneol	1.79
α-humulene	0.97			Verbenone	0.04
Benzyl benzoate	1.43			Geranial	0.02
Caryophyllene oxide	0.64			δ-cadinene	0.08
				y-cadinene	0.04
				Geraniol	0.06
				p-cymene-8-olo	0.06
				Thymol	47.58
				Iso-carvacrol	0.11
				Carvacrol	2.79

**Table 2:** Details of the experimentation carried out during the consolidation of wooden poles.

Tank Name	Tank Feature	Biocide
V2	Transparent	Preventol^®^ RI80
V3	Opaque	Preventol^®^ RI80
V4	Opaque	Cinnamon essential oil
V5	Transparent	Cinnamon essential oil

**Table 3:** Estimation of the minimum inhibitory concentration (MIC) of the EOs. +: presence of growth; -: absence of growth.

Essential Oil	Concentration	*Chaetomium* sp.	*Fusarium* sp.	*Aspergillus japonicus*	*Stachybotrys chartarum*
C	3%	-	-	-	-
	1%	-	-	-	-
	0.7%	+	+	-	+
	0.5%	-	-	+	-
WT	3%	-	-	-	-
	1%	-	-	-	-
	0.7%	-	+	+	-
	0.5%	+	+	+	-
CT	3%	-	-	-	-
	1%	-	-	-	-
	0.7%	-	+	+	-
	0.5%	+	+	+	-

**Table 4:** Evaluation of the biocidal activity of the EOs MIC on fungal mycelia through ATP bioluminescence assay. (C: cinnamon; WT: wild thyme; CT: common thyme; WE: water-ethanol; PT: pretreatment, AT: after treatment).

		*Chaetomium* sp.	*Fusarium* sp.	*Aspergillus japonicus*	*Stachybotrys chartarum*
C	PT (RLU)	141,463,153	238,026,707	40,249,203	101,396,802
	AT (RLU)	8,370	4,700	2,857	26,069
	% decrease	99.9	100	99.9	100
WT	PT (RLU)	63,859,443	184,490,878	28,196,658	225,239,286
	AT (RLU)	0	16,528	7,225	370,328
	% decrease	100	99.9	99.9	100
CT	PT (RLU)	74,999,796	94,931,472	53,213,664	70,763,254
	AT (RLU)	0	4,707	4,413	1,234,871
	% decrease	100	100	99.9	98
WE	PT (RLU)	64,789,488	125,084,954	21,664,705	207,549,160
	AT (RLU)	41,098	179,711,160	13,645,291	13,886,664
	% decrease	99.9	−43.6	37	93

**Table 5:** Evaluation of the biocidal activity of the EOs MIC through ATP bioluminescence assays carried out on storage water and on WAW samples. % decrease was calculated comparing the ATP values obtained for the different treatments with not-treated control (C: cinnamon; WT: wild thyme; CT: common thyme; E: ethanol; NT: not-treated control).

		Water	Wood
C	RLU	0	3,081
	% decrease	100	99.9
WT	RLU	62,212	133,924
	% decrease	89	96.7
CT	RLU	152,611	61,107
	% decrease	73.1	98.5
E	RLU	6,389,944	4,757,930
	% decrease	−1,023.8	−15.2
NT	RLU	568,597	4,130,445

**Table 6:** Evaluation of the biocidal activity of Preventol^®^ RI80 and cinnamon essential oil (C) through bioluminescence assays. (PT: pretreatment, AT: after treatment).

		Cellular ATP (RLU)	
Tank	Biocide	PT	AT
V2	Preventol^®^ RI80	1,134,529	307
V3	Preventol^®^ RI80	6,183,092	16,118
V4	C	1,123,270	11,553
V5	C	2,431,215	23,251

**Table 7:** Evaluation of the biocidal activity of Preventol^®^ RI80 and cinnamon essential oil (C) through microbiological analyses. (PT: pretreatment, AT: after treatment).

		CFU/mL	
Tank	Biocide	PT	AT
V2	Preventol^®^ RI80	6,100,000	4
V3	Preventol^®^ RI80	4,160,000	-
V4	C	32,000,000	6,000
V5	C	9,200,000	120,600

**Table 8:** Percent decrease of the amount of ATP and colony forming units (CFU) after the biocidal treatment.

Tank	Biocide	ATP Decrease (%)	CFU Decrease (%)
V2	Preventol^®^ RI80	99.9	99.9
V3	Preventol^®^ RI80	99.7	100
V4	C	98.9	99.9
V5	C	99	98.6

## Supplementary Materials

The following are available online at https://www.mdpi.com/2076-2607/8/12/2015/s1, Table S1. Raw data obtained for 16S and ITS, Figure S1. Stacked barcharts showing the relative abundance of bacterial classes in each sample. Tips of the clustering tree are colored according to the treatments. (C: cinnamon; E: ethanol; CT: common thyme; NT: not-treated control; WT: wild thyme), Figure S2. Stacked barcharts showing the relative abundance of bacterial orders in each sample. Tips of the clustering tree are colored according to the treatments. (C: cinnamon; E: ethanol; CT: common thyme; NT: not-treated control; WT: wild thyme), Figure S3. Stacked barcharts showing the relative abundance of bacterial genera in each sample. Tips of the clustering tree are colored according to the treatments. (C: cinnamon; E: ethanol; CT: common thyme; NT: not-treated control; WT: wild thyme), Figure S4. Boxplots showing the relative abundance of selected bacterial taxa identified as biomarkers at the LEfSe analysis. (C: cinnamon; E: ethanol; CT: common thyme; NT: not-treated control; WT: wild thyme), Figure S5. Stacked barcharts showing the relative abundance of fungal classes in each sample. Tips of the clustering tree are colored according to the treatments. (C: cinnamon; E: ethanol; CT: common thyme; NT: not-treated control; WT: wild thyme), Figure S6. Stacked barcharts showing the relative abundance of fungal families in each sample. Tips of the clustering tree are colored according to the treatments. (C: cinnamon; E: ethanol; CT: common thyme; NT: not-treated control; WT: wild thyme), Figure S7. Stacked barcharts showing the relative abundance of fungal orders in each sample. Tips of the clustering tree are colored according to the treatments. (C: cinnamon; E: ethanol; CT: common thyme; NT: not-treated control; WT: wild thyme), Figure S8. Stacked barcharts showing the relative abundance of fungal genera in each sample. Tips of the clustering tree are colored according to the treatments. (C: cinnamon; E: ethanol; CT: common thyme; NT: not-treated control; WT: wild thyme), Figure S9. Effect of cinnamon essential oil on one of the pumps used for bath recirculation during the consolidation of archaeological wooden poles.
