# Supplementary material for: Essential Oils as Alternative Biocides for the Preservation of Waterlogged Archaeological Wood
Source: Microorganisms. 2020 Dec 16;8(12):2015. doi: 10.3390/microorganisms8122015 (PMC7765822; doi:10.3390/microorganisms8122015)
Supplement: Supplementary file 1 [file microorganisms-08-02015-s001.zip › Antonelli et al_revised supplementary material.docx]

Article

Essential oils as alternative biocides for the preservation of waterlogged archaeological wood

Federica Antonelli^1,^*, Marco Bartolini^2^, Marie-Laure Plissonnier^3^, Alfonso Esposito^4^, Giulia Galotta^2^, Sandra Ricci^2^, Barbara Davidde Petriaggi^5^, Cristian Pedone^6^, Antonella Di Giovanni^6^, Silvano Piazza^4,7^, Francesca Guerrieri^3,^*, and Manuela Romagnoli^1^

**Supplementary materials**

**Table S1.** Raw data obtained for 16S and ITS.

| **Sample ID** | **Type** | **Treatment** | **16S Sequence Count** | **ITS Sequence Count** | **16S OTU Count** | **ITS OTU Count** |
| --- | --- | --- | --- | --- | --- | --- |
| B1 | Wood | Untreated-control | 28,233 | 107,982 | 445 | 42 |
| B2 | Wood | Ethanol-control | 95,198 | 23,361 | 867 | 89 |
| B3 | Wood | Cinnamon | 115,387 | 11,324 | 690 | 61 |
| B4 | Wood | Wild thyme | 177,514 | 8,725 | 699 | 41 |
| B5 | Wood | Common thyme | 108,162 | 9,961 | 574 | 73 |
| S6 | Storage water | Untreated-control | 74,538 | 17,021 | 395 | 26 |
| S7 | Storage water | Ethanol-control | 148,779 | 5,515 | 1,106 | 61 |
| S8 | Storage water | Cinnamon | 81,521 | 2,857 | 545 | 37 |
| S9 | Storage water | Wild thyme | 180,107 | 3,363 | 292 | 24 |
| S10 | Storage water | Common thyme | 129,798 | 2,809 | 321 | 74 |
| B11 | Wood | Untreated-control | 60,854 | 199,034 | 514 | 39 |
| B12 | Wood | Untreated-control | 117,241 | 154,038 | 742 | 50 |
| B13 | Wood | Ethanol-control | 33,457 | 45,269 | 565 | 130 |
| B14 | Wood | Ethanol-control | 138,146 | 102,693 | 1,095 | 98 |
| B15 | Wood | Wild thyme | 57,800 | 54,154 | 410 | 77 |
| B16 | Wood | Wild thyme | 70,995 | 46,104 | 466 | 62 |
| B17 | Wood | Common thyme | 51,652 | 23,481 | 415 | 87 |
| B18 | Wood | Common thyme | 49,146 | 36,917 | 521 | 86 |
| B19 | Wood | Cinnamon | 50,906 | 17,609 | 547 | 77 |
| B20 | Wood | Cinnamon | 84,278 | 20,502 | 688 | 78 |
|  |  |  |  |  |  |  |

**Table S2.** Diversity values and t-test on the distributions of Shannon diversity values across treatments.

| **Label** | **Type** | **Treatment** | **Replicate** | **Shannon** | **t-test** | **p-values** |
| --- | --- | --- | --- | --- | --- | --- |
| B19 | Wood | Cinnamon | 2 | 2.56 | C Vs CT | 0.016 |
| B20 | Wood | Cinnamon | 3 | 2.87 | C Vs WT | 0.0975978 |
| B3 | Wood | Cinnamon | 1 | 2.83 | C Vs NT | 0.0498253 |
| S8 | Storage water | Cinnamon | 1 | 2.57 |  |  |
| B13 | Wood | Ethanol-control | 2 | 3.13 |  |  |
| B14 | Wood | Ethanol-control | 3 | 3.21 |  |  |
| B2 | Wood | Ethanol-control | 1 | 3.12 |  |  |
| S7 | Storage water | Ethanol-control | 1 | 2.51 |  |  |
| B17 | Wood | Common thyme | 2 | 2.31 |  |  |
| B18 | Wood | Common thyme | 3 | 2.45 |  |  |
| B5 | Wood | Common thyme | 1 | 2.36 |  |  |
| S10 | Storage water | Common thyme | 1 | 1.99 |  |  |
| B1 | Wood | Untreated-control | 1 | 2.24 |  |  |
| B11 | Wood | Untreated-control | 2 | 1.60 |  |  |
| B12 | Wood | Untreated-control | 3 | 1.75 |  |  |
| S6 | Storage water | Untreated-control | 1 | 1.89 |  |  |
| B15 | Wood | Wild thyme | 2 | 2.15 |  |  |
| B16 | Wood | Wild thyme | 3 | 2.70 |  |  |
| B4 | Wood | Wild thyme | 1 | 2.41 |  |  |
| S9 | Storage water | Wild thyme | 1 | 1.56 |  |  |


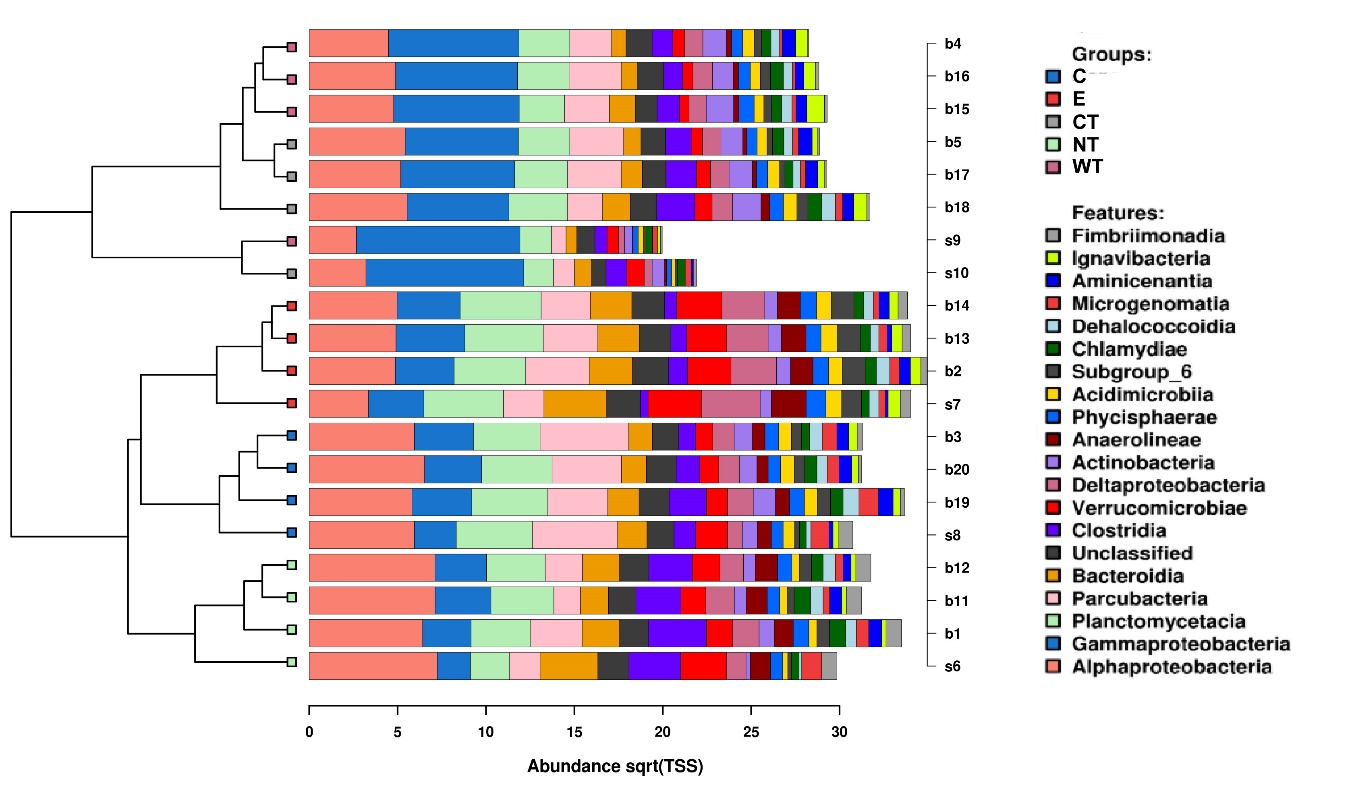


**Figure S1.** Stacked barcharts showing the relative abundance of bacterial classes in each sample. Tips of the clustering tree are coloured according to the treatments. (C: cinnamon; E: ethanol; CT: common thyme; NT: not-treated control; WT: wild thyme).


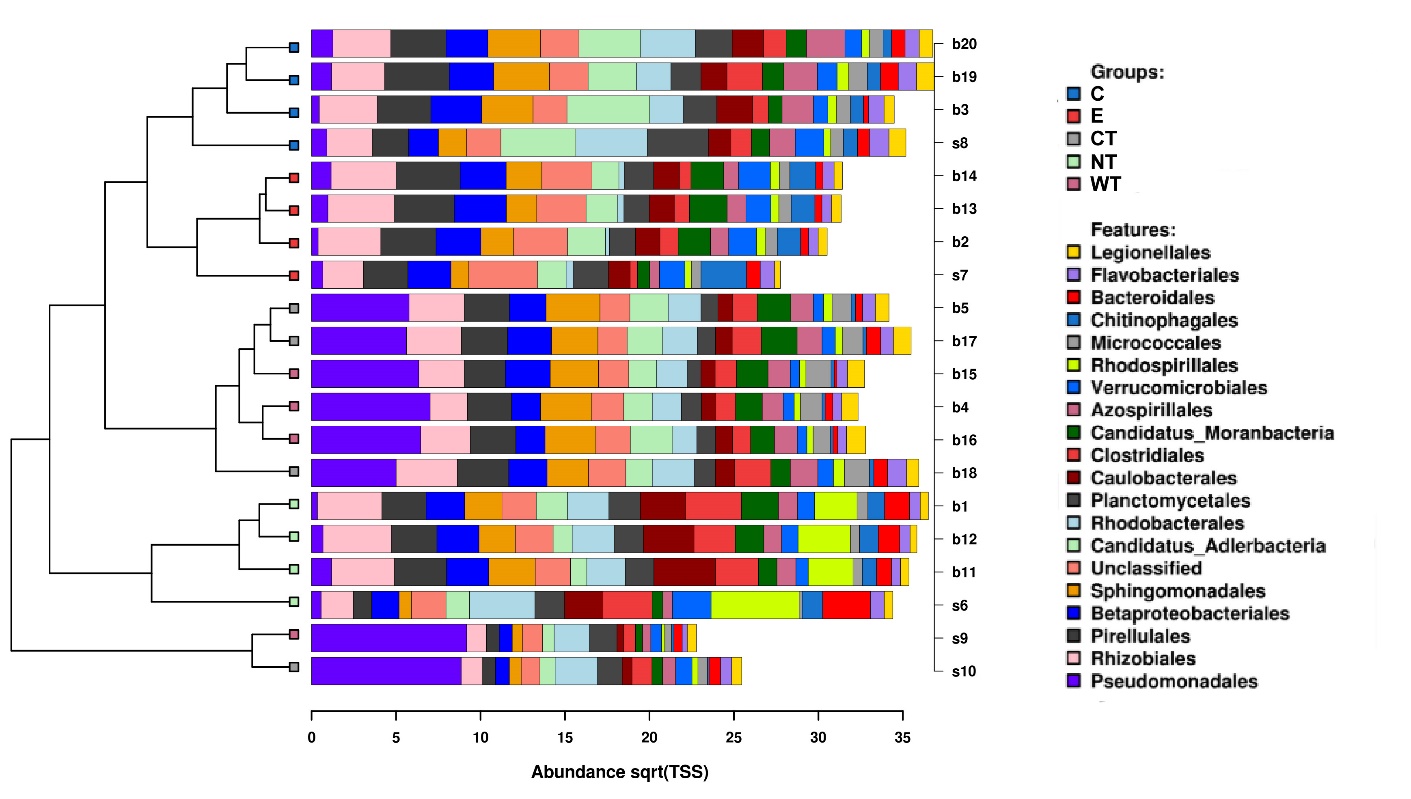


**Figure S2.** Stacked barcharts showing the relative abundance of bacterial orders in each sample. Tips of the clustering tree are coloured according to the treatments. (C: cinnamon; E: ethanol; CT: common thyme; NT: not-treated control; WT: wild thyme).


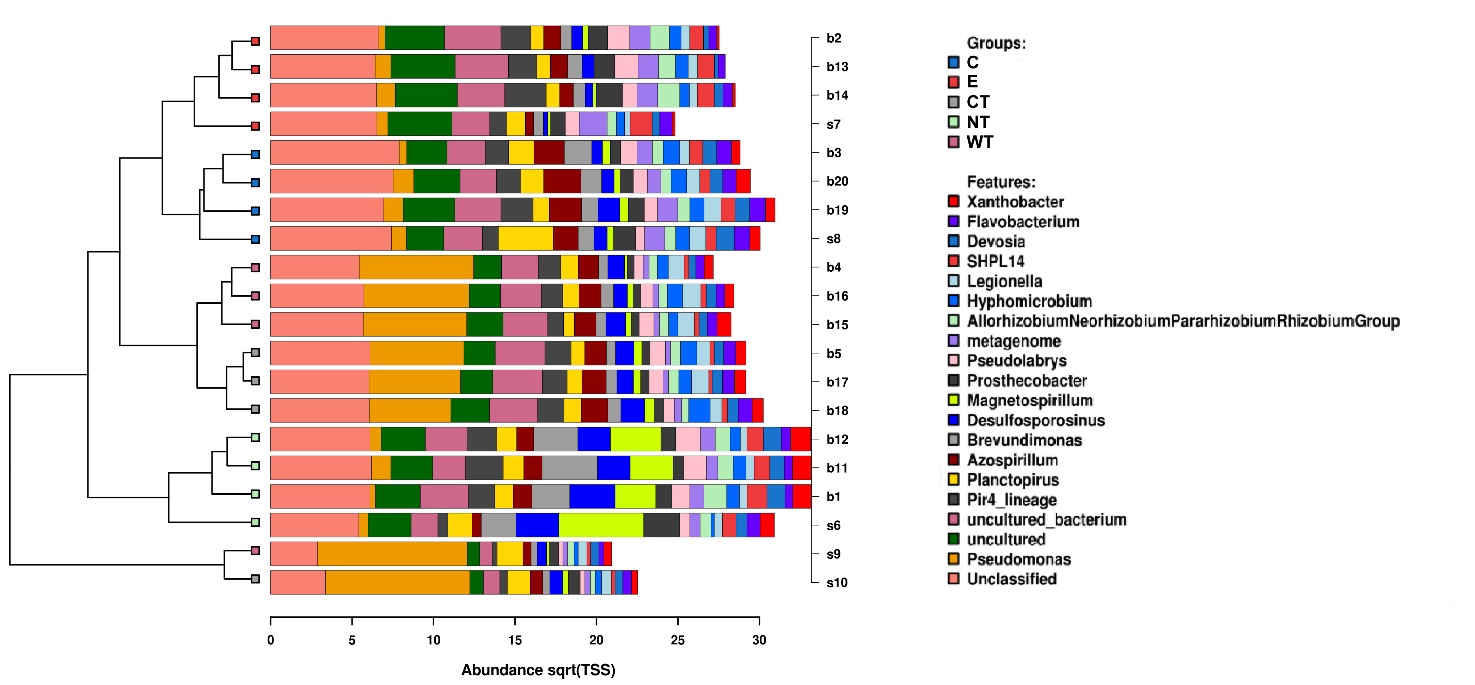


**Figure S3.** Stacked barcharts showing the relative abundance of bacterial genera in each sample. Tips of the clustering tree are coloured according to the treatments. (C: cinnamon; E: ethanol; CT: common thyme; NT: not-treated control; WT: wild thyme).


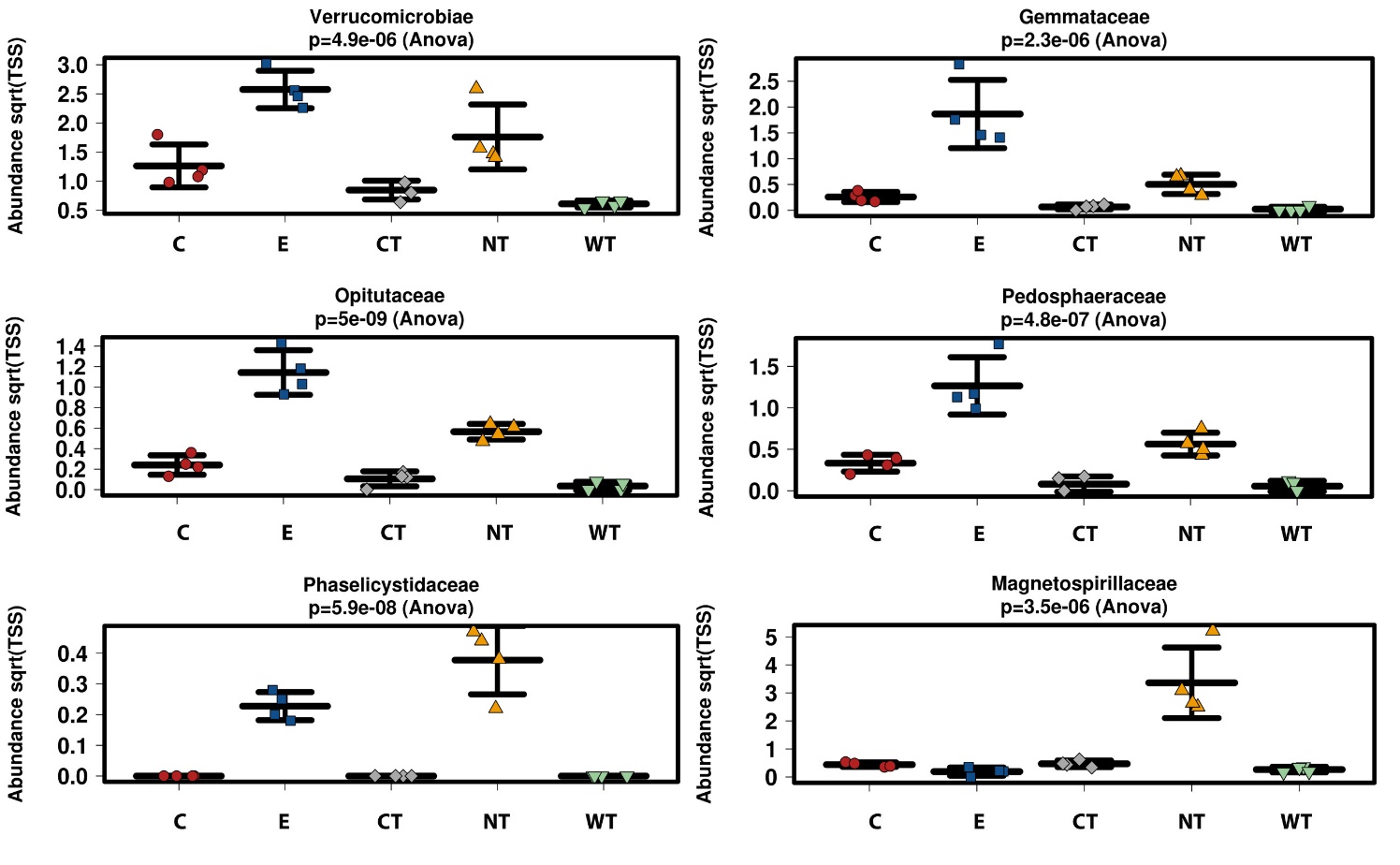


**Figure S4**. Boxplots showing the relative abundance of selected bacterial taxa identified as biomarkers at the LEfSe analysis. (C: cinnamon; E: ethanol; CT: common thyme; NT: not-treated control; WT: wild thyme).


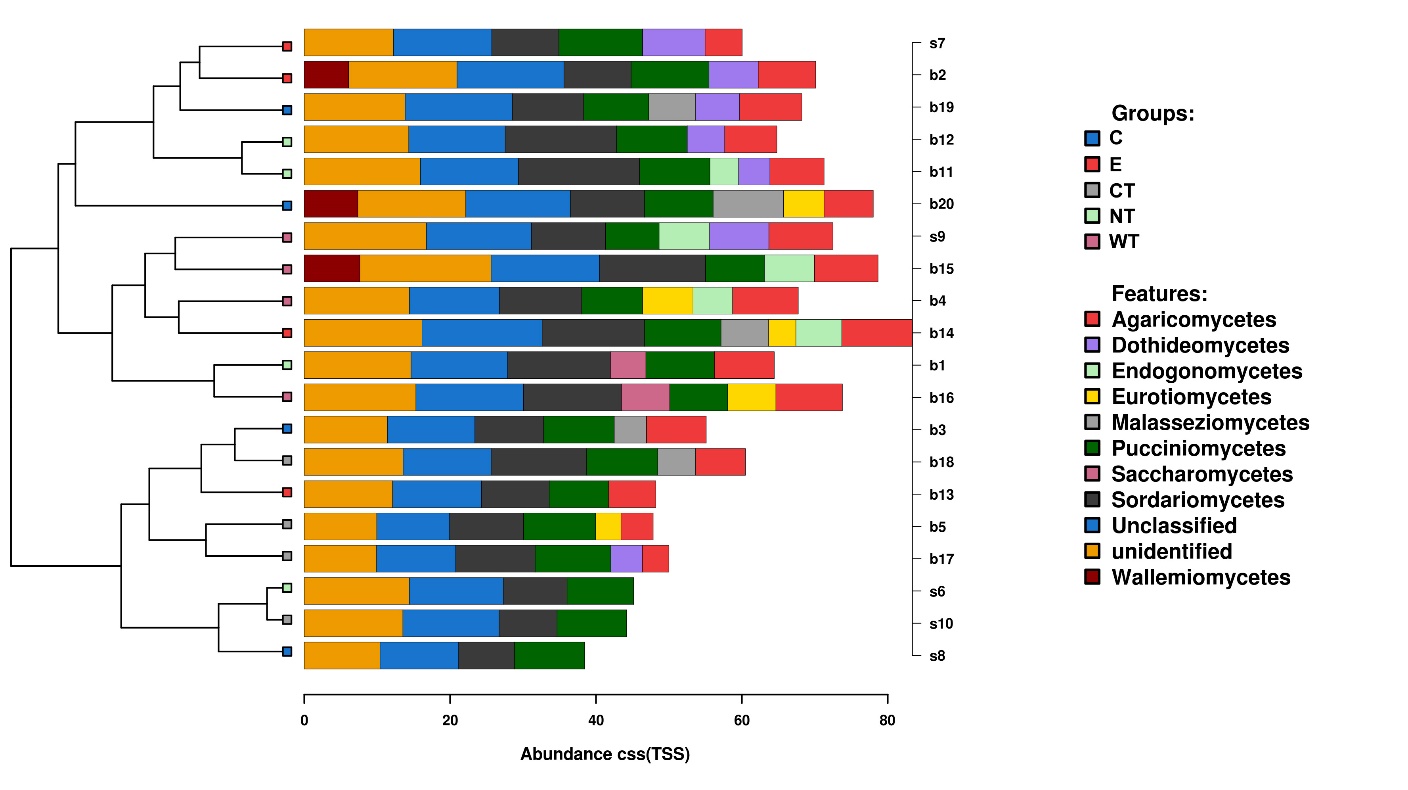


**Figure S5.** Stacked barcharts showing the relative abundance of fungal classes in each sample. Tips of the clustering tree are coloured according to the treatments. (C: cinnamon; E: ethanol; CT: common thyme; NT: not-treated control; WT: wild thyme).


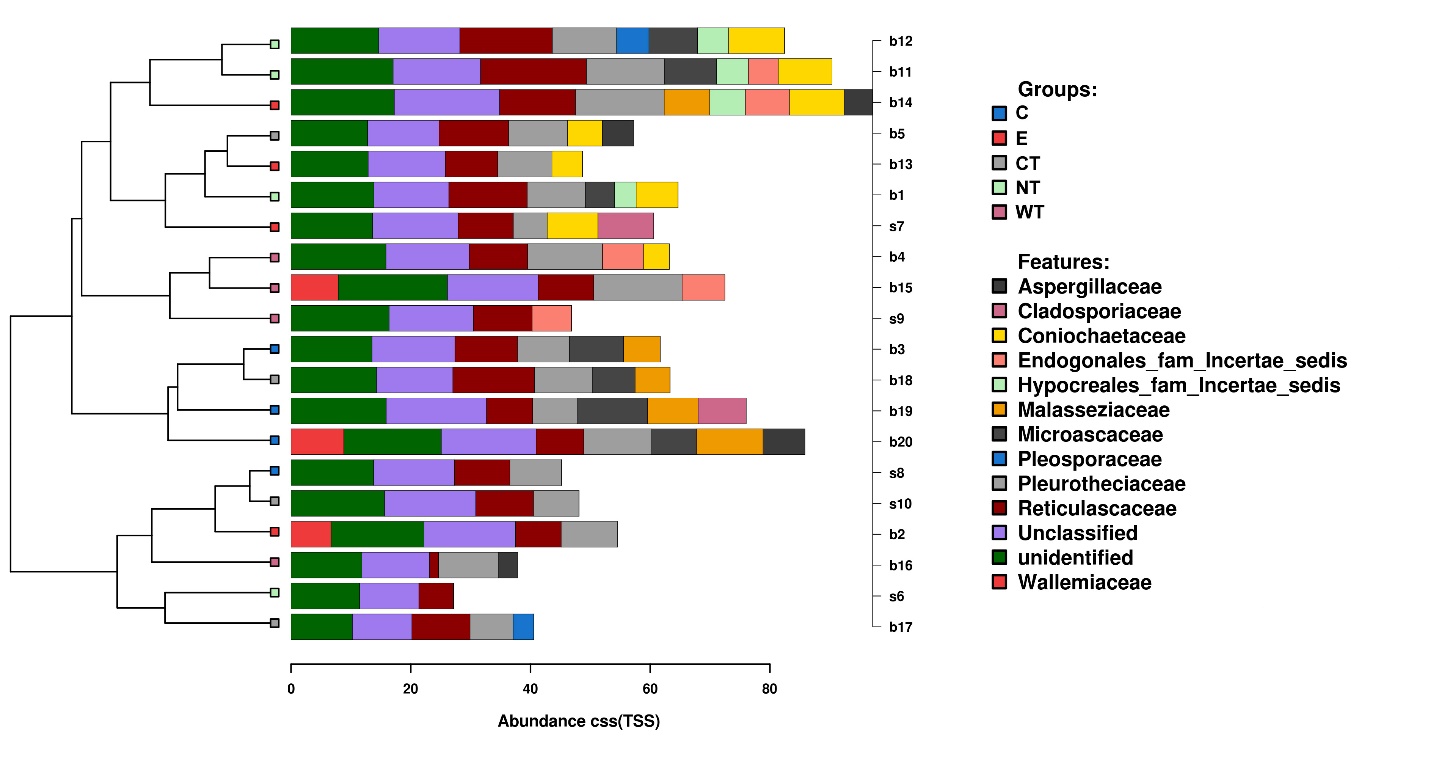


**Figure S6.** Stacked barcharts showing the relative abundance of fungal families in each sample. Tips of the clustering tree are coloured according to the treatments. (C: cinnamon; E: ethanol; CT: common thyme; NT: not-treated control; WT: wild thyme).


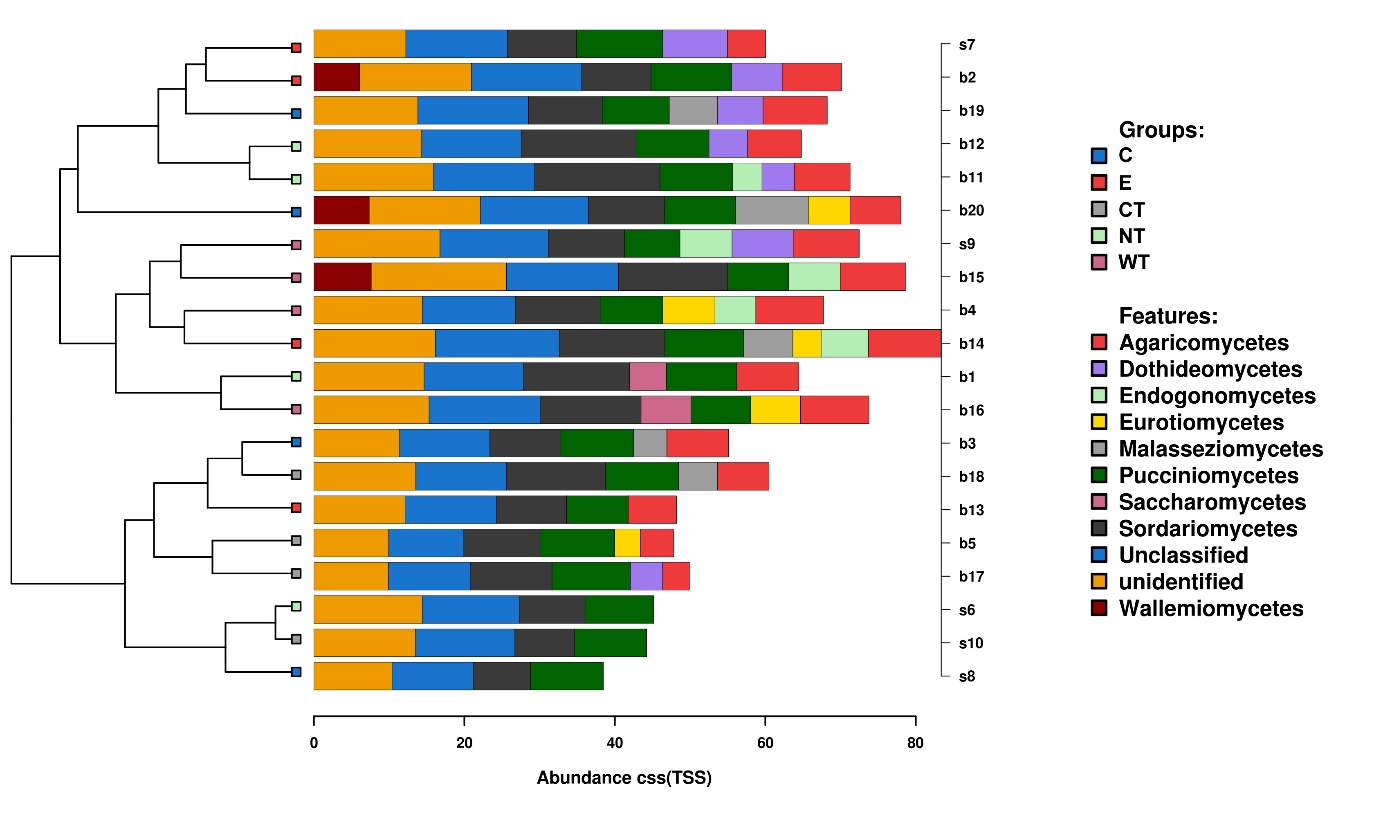


**Figure S7.** Stacked barcharts showing the relative abundance of fungal orders in each sample. Tips of the clustering tree are coloured according to the treatments. (C: cinnamon; E: ethanol; CT: common thyme; NT: not-treated control; WT: wild thyme).


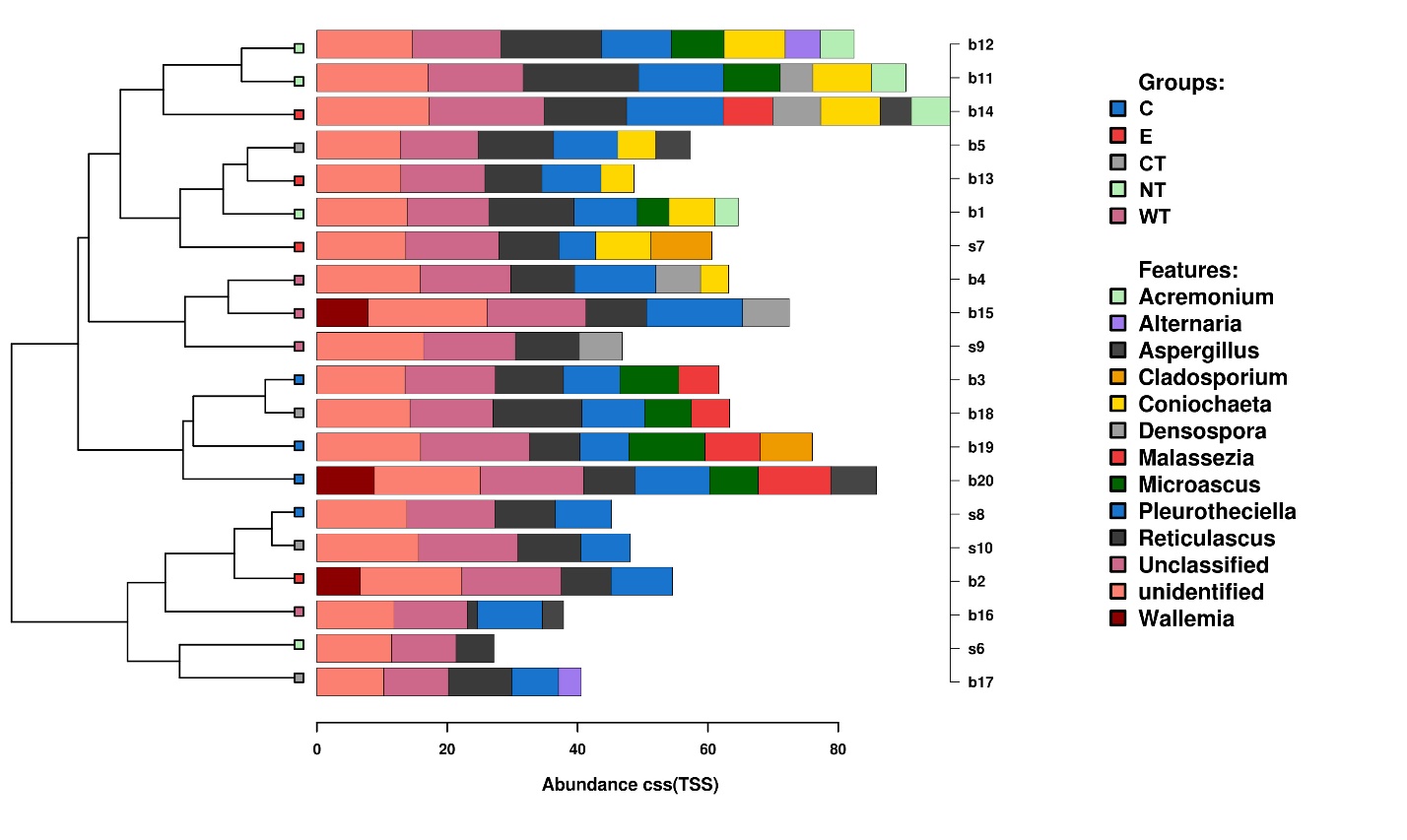


**Figure S8.** Stacked barcharts showing the relative abundance of fungal genera in each sample. Tips of the clustering tree are coloured according to the treatments. (C: cinnamon; E: ethanol; CT: common thyme; NT: not-treated control; WT: wild thyme).


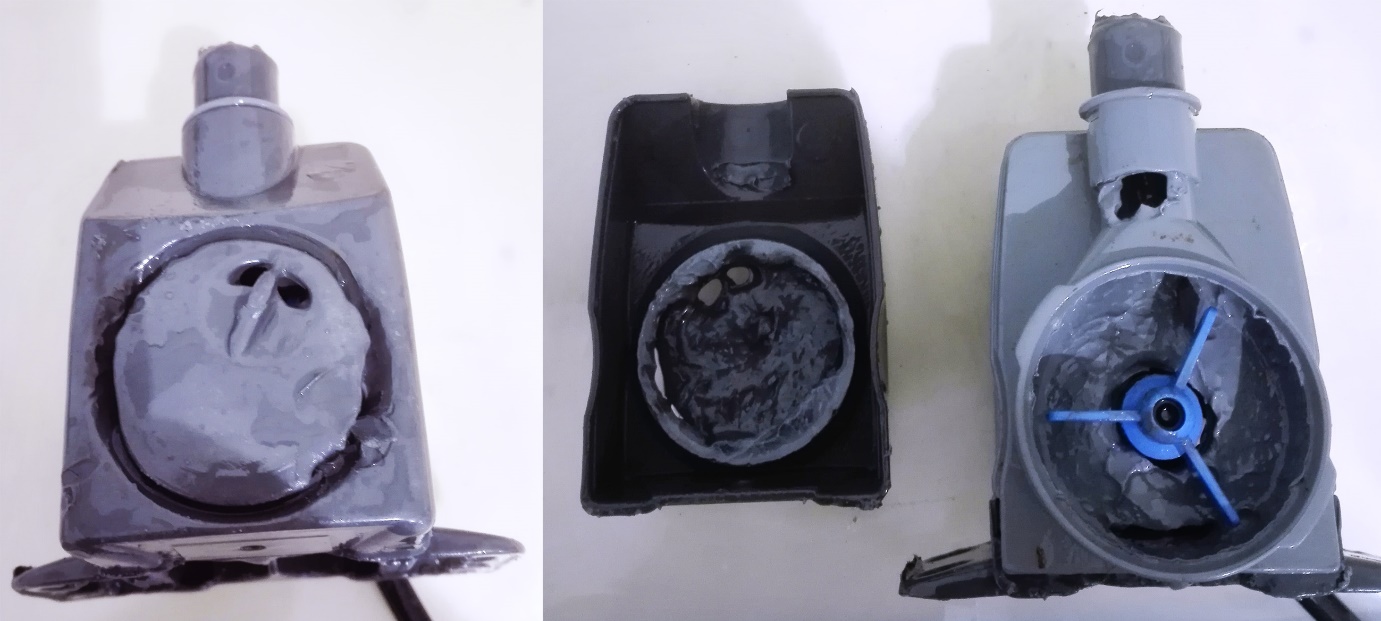


**Figure S9.** Effect of cinnamon essential oil on one of the pumps used for bath recirculation during the consolidation of archaeological wooden poles.

| 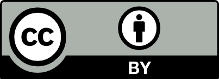 | © 2020 by the authors. Submitted for possible open access publication under the terms and conditions of the Creative Commons Attribution (CC BY) license (http://creativecommons.org/licenses/by/4.0/). |
| --- | --- |
