# Supplementary figures and images for: Essential Oils as Alternative Biocides for the Preservation of Waterlogged Archaeological Wood
Source: Microorganisms. 2020 Dec 16;8(12):2015. doi: 10.3390/microorganisms8122015 (PMC7765822; doi:10.3390/microorganisms8122015)

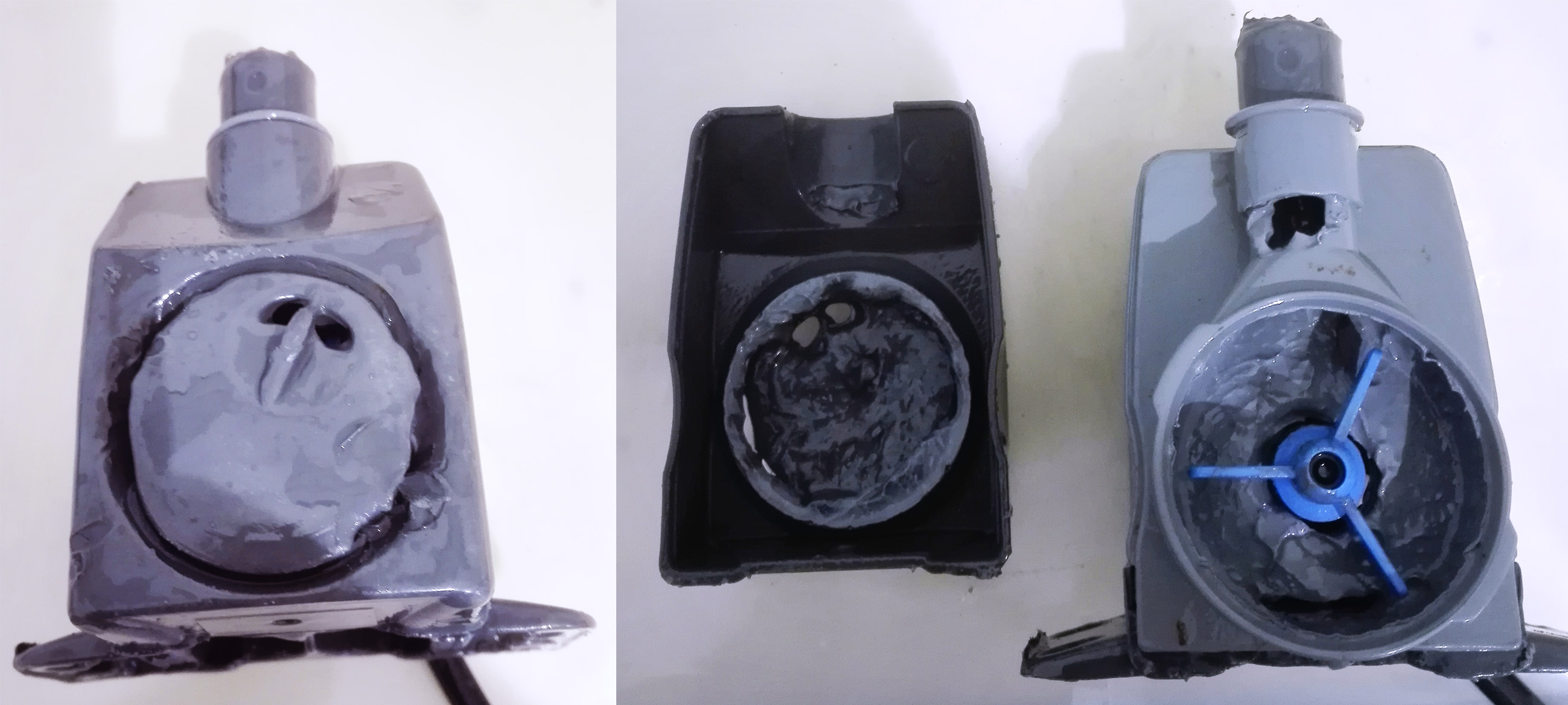

Supplement: Supplementary file 1 [file microorganisms-08-02015-s001.zip › Figure S9.jpg]

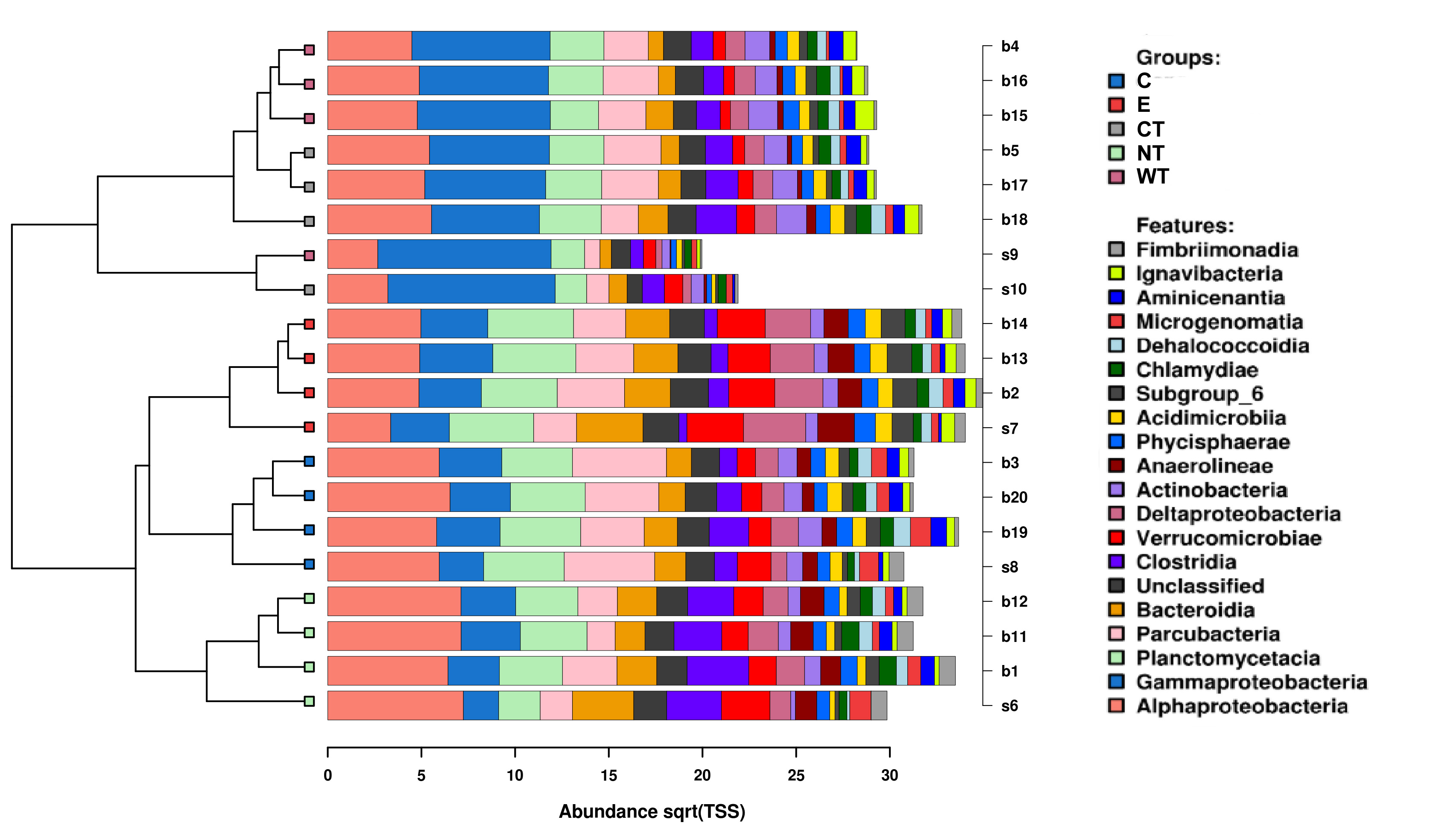

Supplement: Supplementary file 1 [file microorganisms-08-02015-s001.zip › Figure S1.jpg]

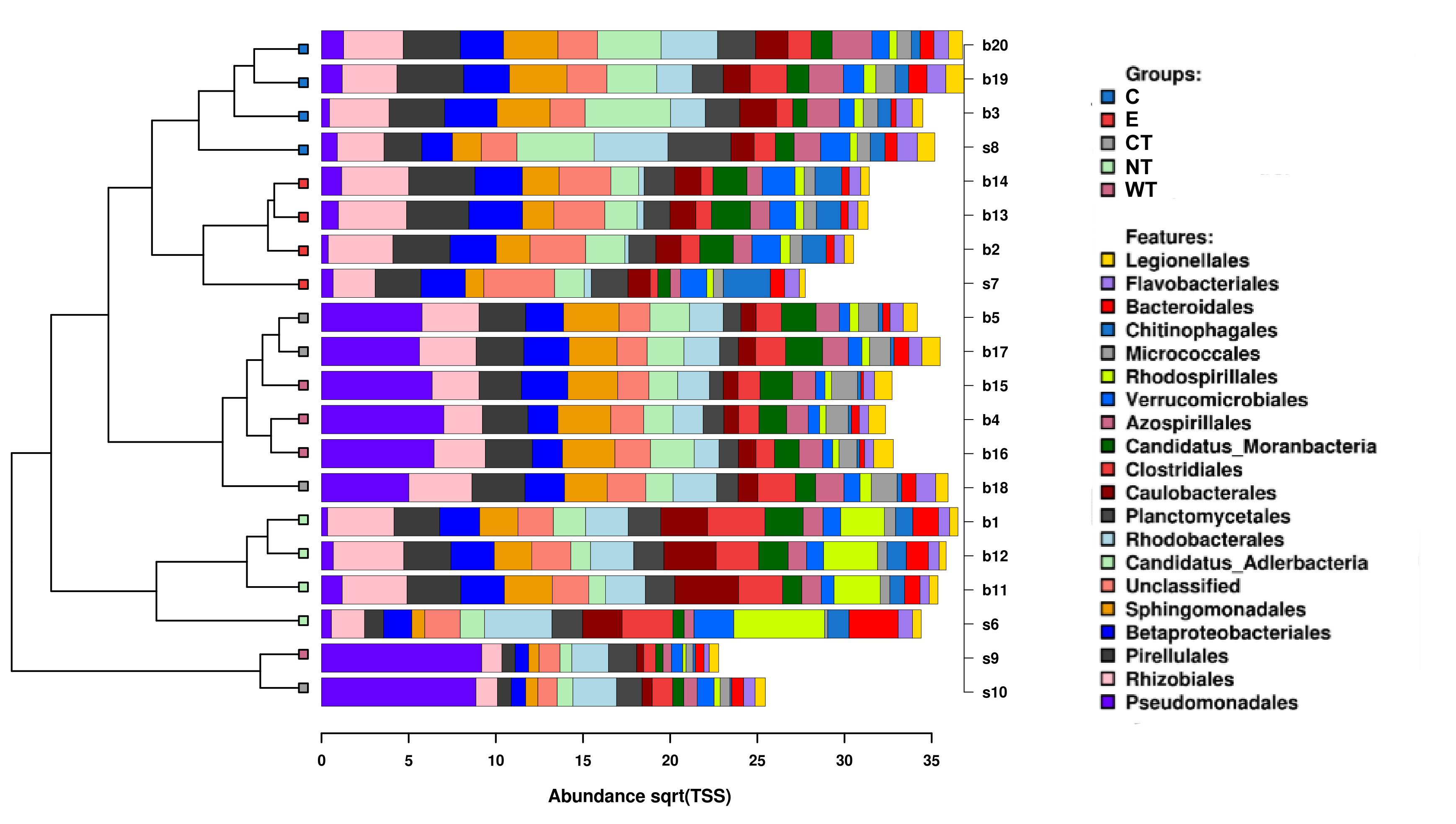

Supplement: Supplementary file 1 [file microorganisms-08-02015-s001.zip › Figure S2.jpg]

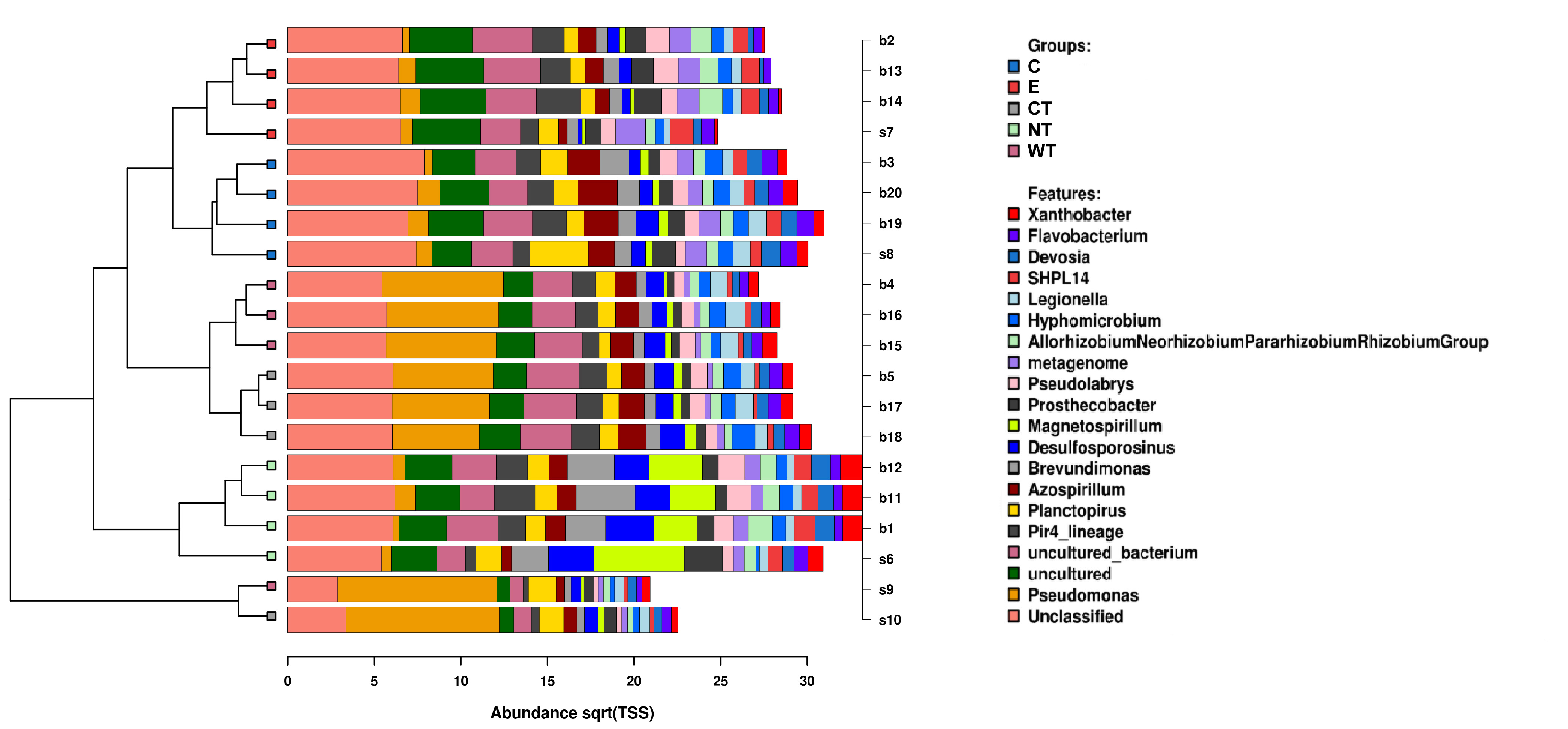

Supplement: Supplementary file 1 [file microorganisms-08-02015-s001.zip › Figure S3.jpg]

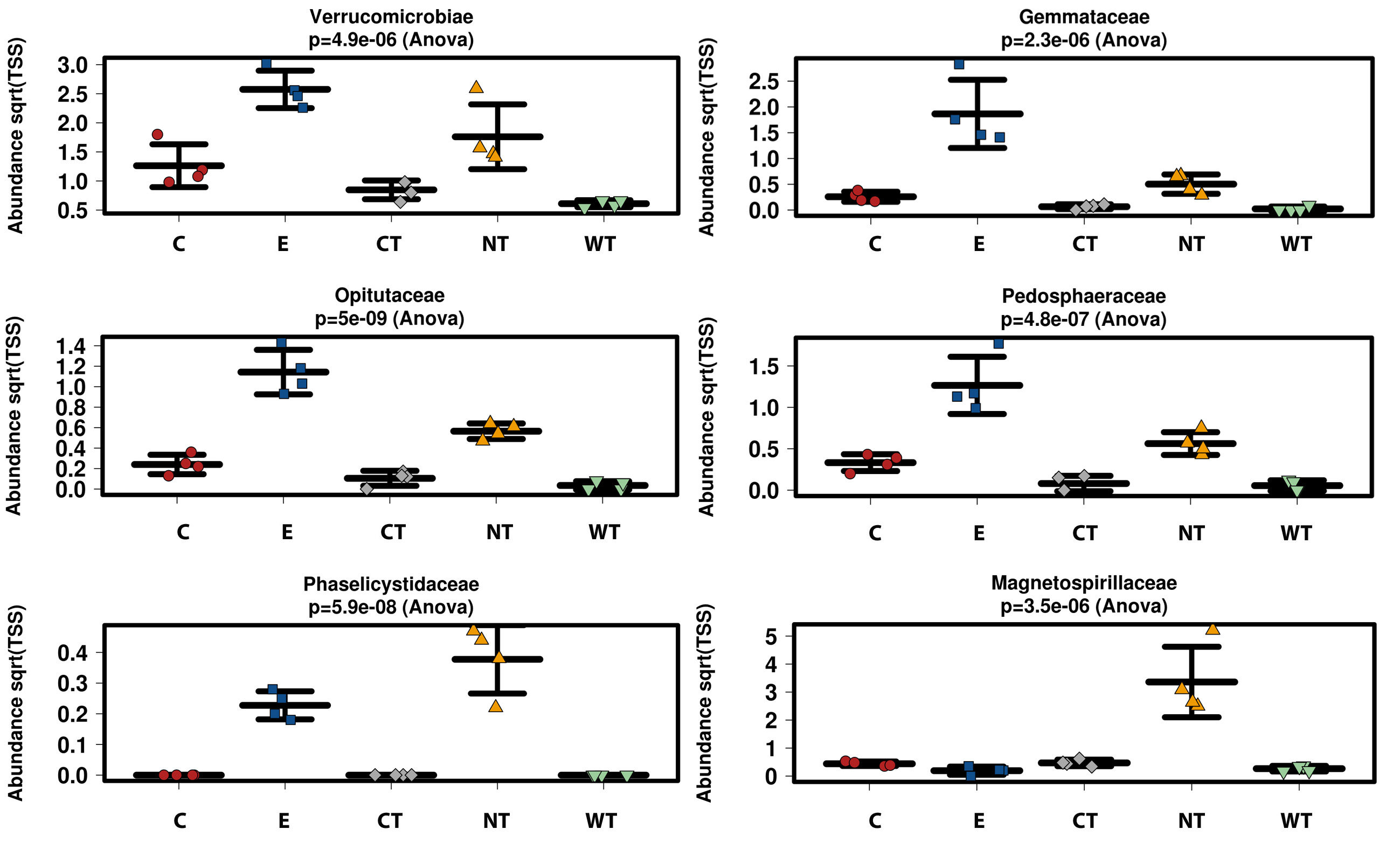

Supplement: Supplementary file 1 [file microorganisms-08-02015-s001.zip › Figure S4.jpg]

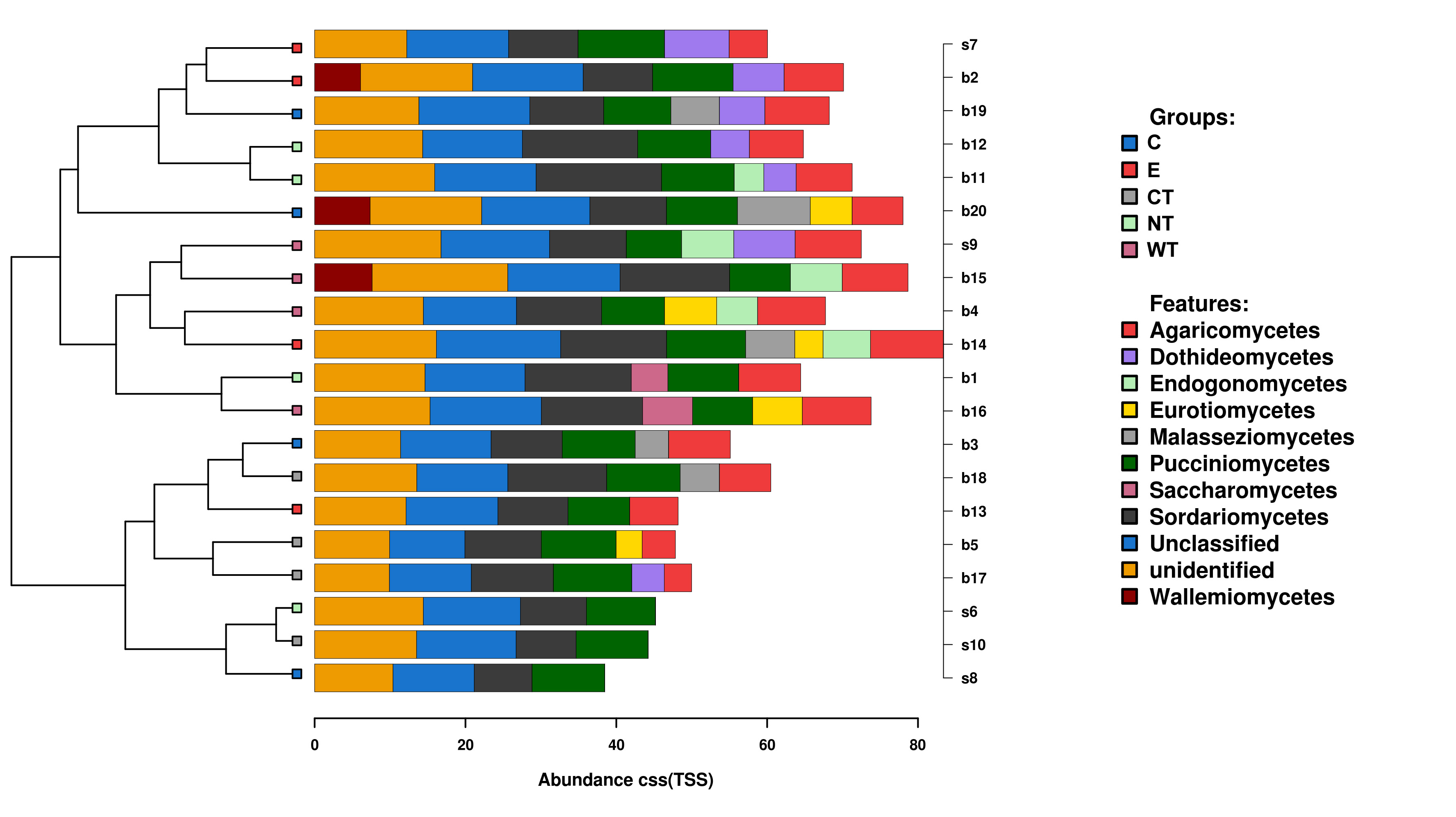

Supplement: Supplementary file 1 [file microorganisms-08-02015-s001.zip › Figure S5.jpg]

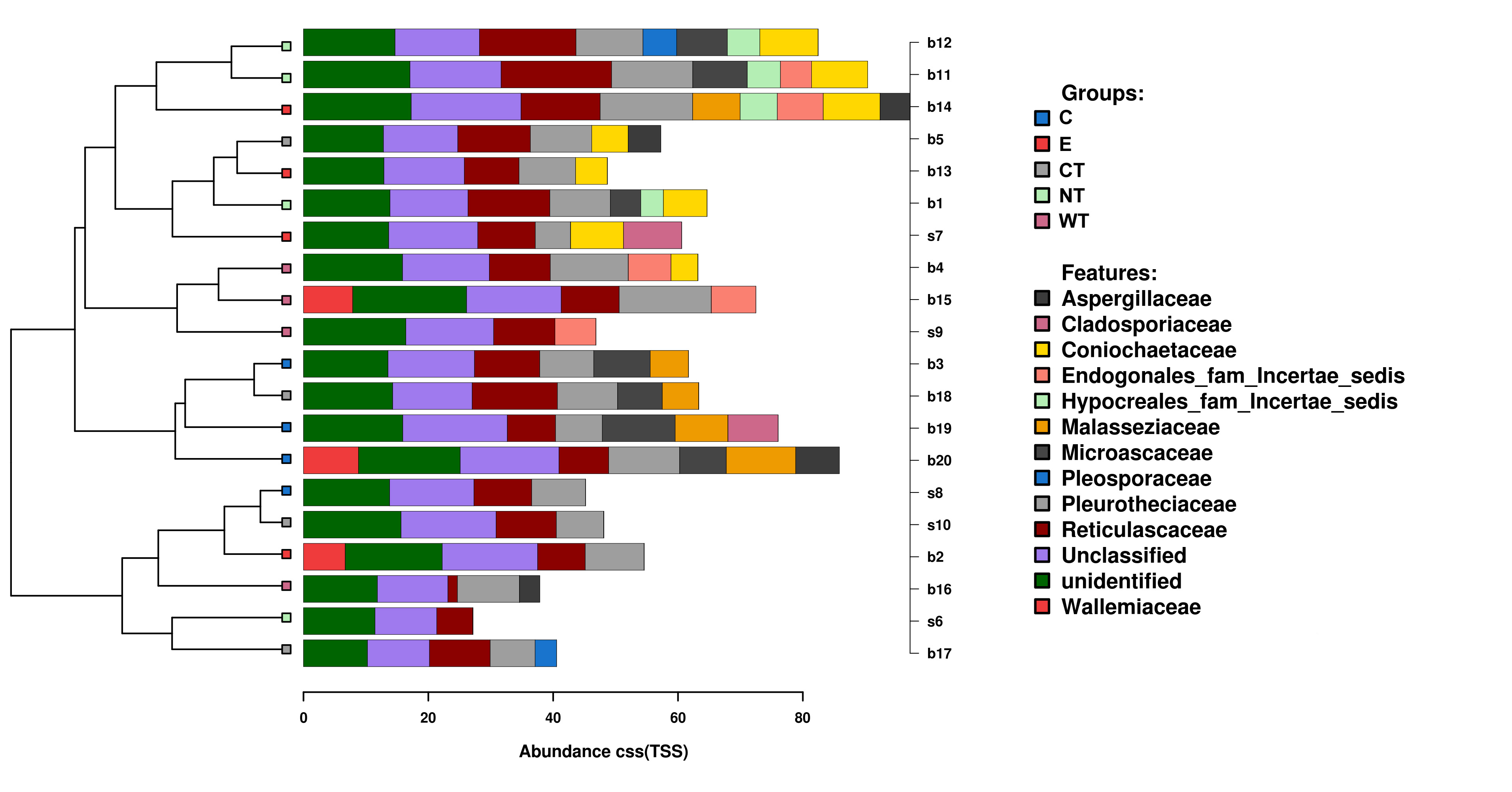

Supplement: Supplementary file 1 [file microorganisms-08-02015-s001.zip › Figure S6.jpg]

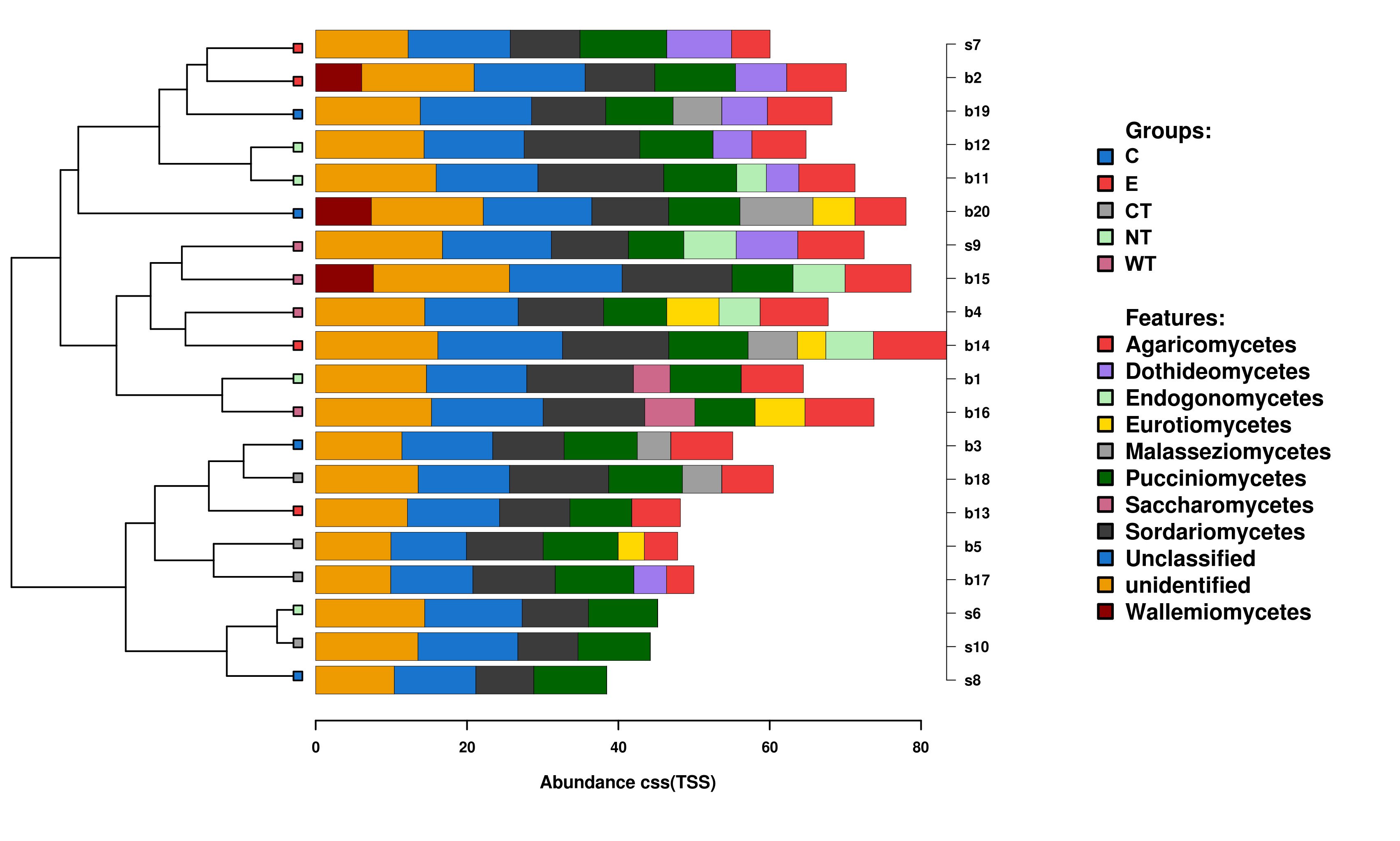

Supplement: Supplementary file 1 [file microorganisms-08-02015-s001.zip › Figure s7.jpg]

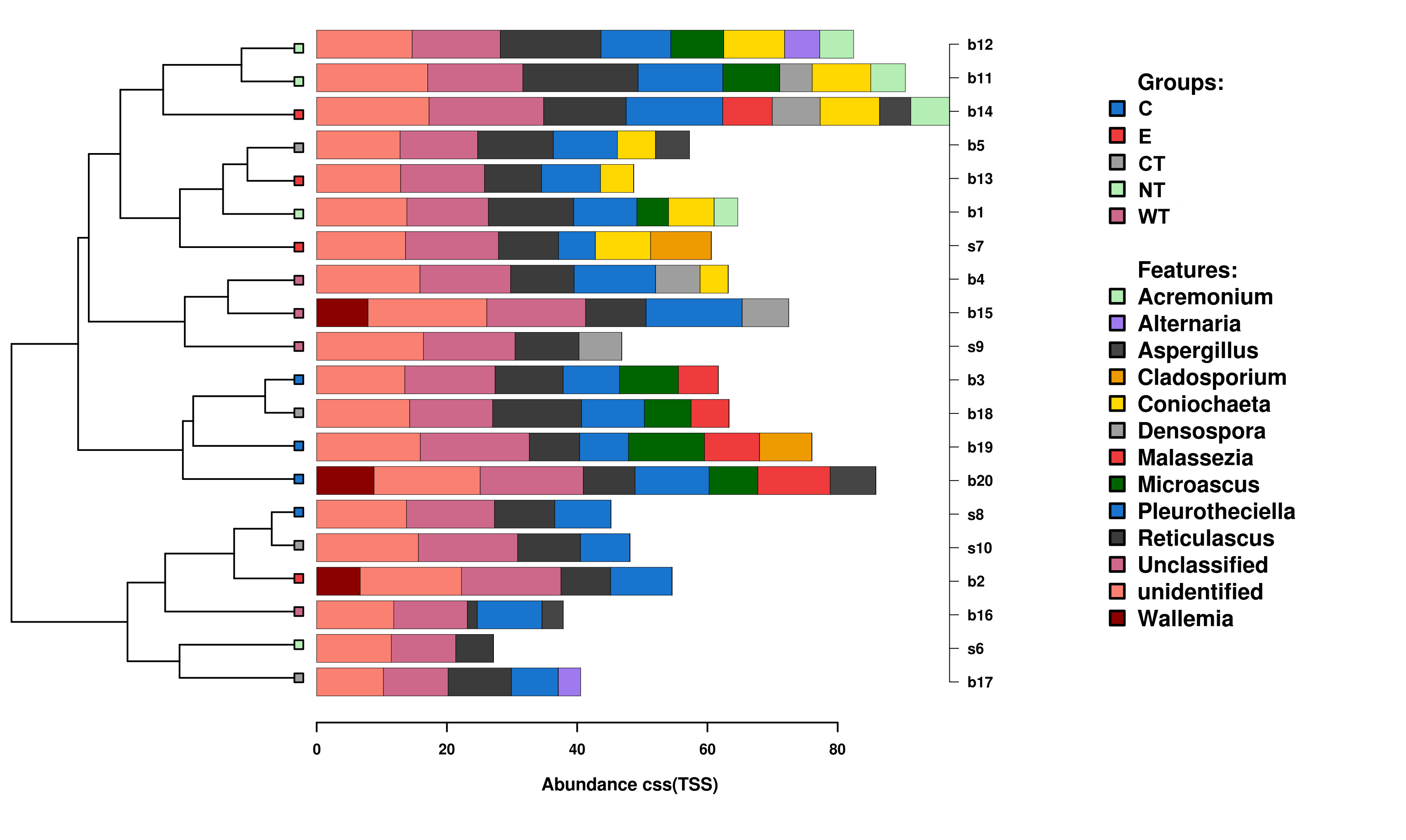

Supplement: Supplementary file 1 [file microorganisms-08-02015-s001.zip › Figure S8.jpg]
